# Polygenic prediction for underrepresented populations through transfer learning by utilizing genetic similarity shared with European populations

**DOI:** 10.1093/bib/bbaf048

**Published:** 2025-02-05

**Authors:** Yiyang Zhu, Wenying Chen, Kexuan Zhu, Yuxin Liu, Shuiping Huang, Ping Zeng

**Affiliations:** Department of Biostatistics, School of Public Health, Xuzhou Medical University, Xuzhou, Jiangsu, 221004, China; Department of Biostatistics, School of Public Health, Xuzhou Medical University, Xuzhou, Jiangsu, 221004, China; Department of Biostatistics, School of Public Health, Xuzhou Medical University, Xuzhou, Jiangsu, 221004, China; Department of Biostatistics, School of Public Health, Xuzhou Medical University, Xuzhou, Jiangsu, 221004, China; Department of Biostatistics, School of Public Health, Xuzhou Medical University, Xuzhou, Jiangsu, 221004, China; Jiangsu Engineering Research Center of Biological Data Mining and Healthcare Transformation, Xuzhou Medical University, Xuzhou, Jiangsu, 221004, China; Department of Biostatistics, School of Public Health, Xuzhou Medical University, Xuzhou, Jiangsu, 221004, China; Jiangsu Engineering Research Center of Biological Data Mining and Healthcare Transformation, Xuzhou Medical University, Xuzhou, Jiangsu, 221004, China

**Keywords:** polygenic score, genome-wide association study, transfer learning, genetic prediction, underrepresented populations

## Abstract

Because current genome-wide association studies are primarily conducted in individuals of European ancestry and information disparities exist among different populations, the polygenic score derived from Europeans thus exhibits poor transferability. Borrowing the idea of transfer learning, which enables the utilization of knowledge acquired from auxiliary samples to enhance learning capability in target samples, we propose transPGS, a novel polygenic score method, for genetic prediction in underrepresented populations by leveraging genetic similarity shared between the European and non-European populations while explaining the trans-ethnic difference in linkage disequilibrium (LD) and effect sizes. We demonstrate the usefulness and robustness of transPGS in elevated prediction accuracy via individual-level and summary-level simulations and apply it to seven continuous phenotypes and three diseases in the African, Chinese, and East Asian populations of the UK Biobank and Genetic Epidemiology Research Study on Adult Health and Aging cohorts. We further reveal that distinct LD and minor allele frequency patterns across ancestral groups are responsible for the dissatisfactory portability of PGS.

## Introduction

The past two decades have witnessed remarkable advances of genome-wide association studies (GWASs) in identifying associated loci (mainly single-nucleotide polymorphisms [SNPs]) for traits and diseases [1–3]. Because most human phenotypes are affected by hundreds or thousands of genetic variants, a single variant typically exerts quite a weak impact compared to traditional nongenetic clinical factors and thereby only explains a very small proportion of phenotypic variation. However, the combination of multiple SNPs weighted by their effect sizes by creating a polygenic score (PGS) usually better reflects the genetic susceptibility to a disease [4–7]. Such a score represents an independent risk factor, which is as equally strong or much stronger than many established clinical risk factors, and has gained great popularity in quantifying an individual’s disease risk [8–12]. It is now widely recognized that PGS, together with clinical and environmental data, can substantially improve the possibility for risk stratification or early disease detection and even pave a road toward personalized intervention [11, 13–17]. As a result, PGS has been extensively utilized to many diseases such as cardiometabolic diseases [8, 13, 15, 17–22].

However, current GWASs have been predominantly conducted in individuals of European (EUR) ancestry, with 94.6% in the EUR population and only 3.7% in the East Asian (EAS) population and 0.2% in the African (AFR) population [23]. Due to this underrepresentation, PGS behaves poorly in non-EUR populations, particularly in populations of AFR ancestry [24–26]. For example, the PGS accuracy reduces ~78% across multiple traits in individuals of AFR ancestry relative to those of EUR ancestry [27]; similarly, the accuracy of PGS across traits is on average 40% lower in individuals of South Asian ancestry and 5% lower in individuals of EAS ancestry compared to those of EUR ancestry [25]. The poor transferability of PGS derived from EUR-ancestry data to non-EUR populations leads to great concern about health disparities [27]. Therefore, there is an urgent need to develop novel PGS methods that can exploit data across diverse populations to better perform genetic risk prediction.

Increasing sample sizes in non-EUR GWASs is a necessary road for the understanding of genetic architecture underlying complex phenotypes of understudied populations such as EAS and AFR, but this requires plenty of expense and time. Alternatively, integrating existing knowledge available from EURs into non-EURs by novel approaches is another promising strategy to promote the portability of PGS. Actually, there is a deal of evidence that significant genetic similarity exists between the EUR and non-EUR populations at both SNP and gene levels [28–33]. Such genetic similarity provides theoretical and biological support for trans-ethnic leveraging of EUR information into non-EUR studies. Currently, there is a range of trans-ethnic statistical methods that help enhance the transferability of PGS across distinct ancestral groups [34]; however, how to more effectively integrate EUR information into non-EUR genetic research remains unknown.

Recently, transfer learning has been applied in various machine learning fields for knowledge transfer from informative auxiliary samples into target samples to improve learning ability in the target task [35–37]. By borrowing this idea, we here propose transPGS, a novel transfer learning genetic prediction method applicable to both individual-level and summary-level GWAS data. Taking the pruning and thresholding (P + T) model as a starting point, transPGS leverages trans-ethnic genetic similarity shared with the EUR population (i.e. auxiliary samples) to adapt the effect sizes in the non-EUR population (i.e. target samples) such as AFR or EAS. Consequently, transPGS is able to aggregate genetic information across distinct populations and improves prediction accuracy, especially in understudied ancestral groups.

To illustrate the effectiveness of transPGS, we conduct extensive simulations and confirm that the predictive ability of transPGS is enhanced in the non-EUR population as the increase of trans-ethnic similarity is shared with the EUR population. Further, with the AFR or EAS population as target samples and the EUR population as auxiliary samples, we also observe improved prediction accuracy in both individual and summary levels when applying transPGS to seven continuous phenotypes and three diseases available from the UK Biobank (UKB) [38] and the Kaiser Permanente/UCSF Genetic Epidemiology of Adult Health and Ageing Study [39, 40]. Overall, through simulations and real data applications, we demonstrate that transPGS represents a flexible and effective polygenic score method, which can improve genetic prediction capability for individuals of non-EUR ancestry.

## Materials and methods

### Individual-level transPGS

#### Genetic prediction models in the target and auxiliary samples

We first describe individual-level transPGS. Let ***Y*** be an *n* × 1 vector of the phenotype (e.g. a binary variable such as disease status, or a continuous variable such as body mass index [BMI]) measured on *n* individuals in the target population, **G** be an *n* × *P* matrix for genotypes of *P* SNPs, and **X** be an *n* × *m* matrix for *m* conventional covariates. We characterize the relation between **X**, **G**, and ***Y*** via a generalized linear mixed model in the target samples


(1) \begin{equation*} g\left(\boldsymbol{\mu} \right)\kern0.33em =\kern0.33em \mathbf{X}\boldsymbol{\alpha} \kern0.33em +\kern0.33em \mathbf{G}\boldsymbol{\beta} \end{equation*}

where *g* denotes the link function, ***μ*** is the mean of ***Y*, *β*** = (${\beta}_1$, …, ${\beta}_P$) is the vector of SNP effect sizes following a normal distribution (i.e. ${\beta}_j$~*N*(0,${\boldsymbol{\sigma}}_g^2$), *j* = 1, …, *P*) [41, 42], **G*β*** is referred to as PGS quantifying genetic influence in the target population, and *α* = (${\alpha}_1$, …, ${\alpha}_m$) is the fixed-effect vector for covariates.

Besides target samples, suppose that we also observe additional samples from the auxiliary population *A_t_* (*t* = 1, …, *T*, with *T* the total number of auxiliary populations), which is informative and provides substantial assistance to estimation and prediction for the target model. In a similar prediction model, we denote the SNP-effect vector of auxiliary samples by ${\boldsymbol{b}}_{\boldsymbol{t}}$ = (${b}_{t1}$, …, ${b}_{tP}$).

#### Utilize genetic information from auxiliary samples to target samples via transfer learning

Since $\boldsymbol{\beta}$ and ${\boldsymbol{b}}_{\boldsymbol{t}}$ are estimated with different samples, they are essentially not identical. To effectively incorporate existing genetic knowledge through a transfer learning manner from the auxiliary samples into the target samples, we assume that there exists a linear connection between these two sets of effect sizes [35, 37, 43, 44].


(2) \begin{equation*} \boldsymbol{\beta} \kern0.33em =\kern0.33em \sum \limits_{t\kern0.33em =\kern0.33em 1}^T\boldsymbol{b}_t{\boldsymbol{\omega}}_t\kern0.33em +\kern0.33em \boldsymbol{\delta} \end{equation*}

where ${\omega}_t$ is a scale parameter, an informative auxiliary study implies that ${\omega}_t$ is significantly different from zero; $\boldsymbol{\delta}$ = (${\delta}_1$, …, ${\delta}_P$) is the vector of target-specific SNP effect sizes following a normal distribution (e.g., ${\delta}_j$~*N*(0,${\boldsymbol{\sigma}}_{\delta}^2$), *j* = 1, …, *P*), which can be also referred to as a discrepancy measuring the inconsistency of effect sizes between the target and auxiliary samples. Inserting the relation (2) into the target model (1), we obtain the transfer learning model


(3) \begin{equation*} g\left(\boldsymbol{\mu} \right)\kern0.33em =\kern0.33em \mathbf{X}\boldsymbol{\alpha} \kern0.33em +\kern0.33em \mathbf{G}\left\{\sum \limits_{t\kern0.33em =\kern0.33em 1}^T{\boldsymbol{b}}_t{\boldsymbol{\omega}}_t\kern0.33em +\kern0.33em \boldsymbol{\delta} \right\}\kern0.33em =\kern0.33em \mathbf{X}\boldsymbol{\alpha} \kern0.33em +\kern0.33em \sum\limits_{t\kern0.33em =\kern0.33em 1}^T\left(\boldsymbol{Gb}_t\right){\boldsymbol{\omega}}_t\kern0.33em +\kern0.33em \mathbf{G}\boldsymbol{\delta} \end{equation*}

where ${\boldsymbol{Gb}}_{{t}}$ can be referred to as the trans-ethnic PGS of target samples with genetic effect sizes estimated directly from auxiliary samples [43].

#### Summary-level transPGS

We now extend individual-level transPGS to the summary-level setting under the framework of summary statistics regression model [45]. Let $\hat{\boldsymbol{\beta}}$=(${\hat{\beta}}_1$, …, ${\hat{\beta}}_P$) be the vector of marginal effect sizes of a set of particularly selected SNPs in the target population and $\hat{\mathbf{S}}$ be a diagonal matrix with its element the standard error of the marginal SNP effect size (i.e., ${\hat{\mathbf{S}}}_{pp}= se\left({\hat{\beta}}_p\right) $, *p* = 1, …, *P*). Within the context of polygenic architecture [46], we obtain the summary statistics regression likelihood


(4) \begin{equation*} LogL\left(\boldsymbol{\beta} |\hat{\boldsymbol{\beta}}\right)\kern0.33em =\kern0.33em -\frac{1}{2}{\left(\hat{\boldsymbol{\beta}}\kern0.33em -\kern0.33em \hat{\mathbf{S}}\mathbf{R}{\hat{\mathbf{S}}}^{-1}\boldsymbol{\beta} \right)}^T{\left({{\boldsymbol{\sigma}}}_e^2\hat{\mathbf{S}}\mathbf{R}\hat{\mathbf{S}}\right)}^{-1}\left(\hat{\boldsymbol{\beta}}\kern0.33em -\kern0.33em \hat{\mathbf{S}}\mathbf{R}{\hat{\mathbf{S}}}^{-1}\boldsymbol{\beta} \right) \end{equation*}

where **R** is the LD matrix, and ${\boldsymbol{\sigma}}_e^2$ is an additional variance applied to explain possible discrepancies including measurement error when calculating LD from external reference panels and potential information loss when using summary-level data rather than individual-level data. When ${\boldsymbol{\sigma}}_e^2$ = 1, it exactly reduces to the regression model of summary statistics given in [45].

It is easy to see that Equation (4) is actually the log-likelihood of a linear model with $\hat{\boldsymbol{\beta}}$ regressing on $\hat{\mathbf{S}}\mathbf{R}{\hat{\mathbf{S}}}^{-\mathbf{1}}$weighted by $\hat{\mathbf{S}}\mathbf{R}\hat{\mathbf{S}}$. Thus, we have an equivalent relation


(5) \begin{equation*} {\boldsymbol{Y}}^{\prime}\kern0.33em =\kern0.33em \boldsymbol{W\beta} \kern0.33em +\kern0.33em \boldsymbol{e},\kern0.33em \boldsymbol{e}\kern0.33em \sim \kern0.33em \mathbf{MVN}\left(0,\kern0.33em {\boldsymbol{\sigma}}_e^2{\boldsymbol{I}}_P\right) \end{equation*}

where ${\boldsymbol{Y}}^{\prime }={\left(\hat{\mathbf{S}}\mathbf{R}\hat{\mathbf{S}}\right)}^{-\frac{1}{2}}\hat{\boldsymbol{\beta}}$, $\mathbf{W}={\left(\hat{\mathbf{S}}\mathbf{R}\hat{\mathbf{S}}\right)}^{-\frac{1}{2}}\hat{\mathbf{S}}\mathbf{R}{\hat{\mathbf{S}}}^{-1}$, and ***e*** represents the residuals. Again, in the similar way, we can construct the summary statistics regression likelihood for ***b****_t_* in the auxiliary population and we utilize the relation (2) to transfer the information of auxiliary samples into target samples


(6) \begin{equation*} {\boldsymbol{Y}}^{\prime}\kern0.33em =\kern0.33em \boldsymbol{W}\sum \limits_{t\kern0.33em =\kern0.33em 1}^T{\omega}_t{b}_t\kern0.33em +\kern0.33em \mathbf{W}\delta \kern0.33em +\kern0.33em \boldsymbol{e},\kern0.33em \boldsymbol{e}\kern0.33em \sim \kern0.33em \mathrm{MVN}\left(0,\kern0.33em {\boldsymbol{\sigma}}_e^2{\boldsymbol{I}}_P\right) \end{equation*}

Details of summary-level transPGS are described in the Supplementary File.

#### Parameter estimation algorithms for transPGS

In individual-level transPGS, we employ the parameter expansion expectation maximization (PX-EM) algorithm [43, 44, 47, 48] to estimate unknown parameters before and after transfer learning for continuous phenotypes and apply the average information–based restricted maximum likelihood (AI-REML) algorithm [49–51] to estimate unknown parameters before and after transfer learning for binary phenotypes. In summary-level transPGS, we utilize the ridge algorithm [52, 53] to estimate unknown parameters in model (5) and use the PX-EM algorithm to estimate unknown parameters in model (6). Further descriptions regarding these algorithms are given in the Supplementary File. To be easily implemented, both individual-level and summary-level transPGS methods follow the two-step estimation procedure commonly employed in multi-omics integrative analysis [37, 43, 44]. R codes conducting transPGS are available at https://github.com/biostatpzeng/transPGS.

### Simulation studies

To evaluate the prediction performance of transPGS, we carried out extensive simulations to generate continuous or binary phenotypes with genotypes available from the UKB cohort [38]. To mimic the real data applications below, in the auxiliary population (with 200 000 randomly selected individuals of EUR descent), we produced the continuous phenotype via a linear model with 1500 common SNPs or created the binary phenotype via a logistic model. These SNPs were obtained from a local region of Chr 1 and had minor allele frequency (MAF) >1%. For continuous phenotypes, an error term following a standardized normal distribution was added. Two independent covariates were also generated in the auxiliary samples (i.e. *Z*_1_ was binary and *Z*_2_ was continuous). The SNP effect sizes (i.e. $\boldsymbol{b}$) were sampled from a normal distribution with a mean zero and a specific variance so that the phenotypic variance explained (PVE) by SNPs was 1%, 5%, or 10%.

The phenotypes of 3000 randomly selected AFR individuals were created in the target population in the similar manner described above; the SNP effect sizes were set to $\boldsymbol{\beta} =\boldsymbol{b}\boldsymbol{\omega } +\boldsymbol{\delta}$, where $\boldsymbol{\delta}$ had a normal distribution with a mean zero and a variance ${\boldsymbol{\sigma}}_{\delta}^2$. We specified $\omega$= 0.1, 0.3, 0.5, 0.7, or 0.9 and ${\boldsymbol{\sigma}}_{\delta}^2$ = 0.01 or 0.04. We repeated 50 simulations for each scenario and evaluated the prediction accuracy by calculating *R*^2^ or the area under the curve (AUC).

To comprehensively evaluate the performance of transPGS, we also conducted simulation studies using genome-wide SNPs. To approach the genome-wide scenario as closely as possible while guaranteeing the computational feasibility of transPGS, we expanded the number of used SNPs to 200 000. These SNPs were randomly selected from 22 chromosomes in an approximately uniform manner. The settings of other parameters remained the same as those in the simulations performed with local genetic variants.

### Real data applications

#### Phenotypes in the UK Biobank and Genetic Epidemiology Research Study on Adult Health and Aging cohorts

We employed transPGS to analyze seven continuous phenotypes from the AFR and Chinese (CHI) populations of the UKB cohort [38] and three diseases from the EAS and AFR populations of the Genetic Epidemiology Research Study on Adult Health and Aging (GERA) cohort [39, 40]. The continuous phenotypes included high-density lipoprotein (HDL), low-density lipoprotein (LDL), total cholesterol (TC), triglyceride (TG), systolic blood pressure (SBP), diastolic blood pressure (DBP), and body mass index (BMI); the diseases included coronary artery disease (CAD), asthma, and type 2 diabetes (T2D). The individuals of EAS, CHI, or AFR ancestry in the UKB or GERA cohort were target samples, while the individuals of EUR ancestry in the UKB or GERA cohort were auxiliary samples (Table 1).

**Table 1:** Sample sizes of the 10 analyzed phenotypes in diverse populations available from the UKB and GERA cohorts.

Phenotype	Target samples		Auxiliary samples
UKB	AFR	CHI	EUR
HDL	6637	1302	402 834
LDL	7159	1424	438 194
TC	7173	1426	438 959
TG	7171	1425	438 616
SBP	7614	1500	458 726
DBP	7614	1500	458 726
BMI	7533	1494	280 575
CAD	1343/5836	/	27 241/422 971
T2D	1183/6446	/	25 069/447 506
Asthma	663/6516	/	56 185/416 402
**GERA**	**AFR**	**EAS**	**EUR**
CAD	1173/2653	816/4372	16 502/45 811
T2D	788/3038	805/4383	7713/54 600
Asthma	690/3136	811/4377	10 151/52 162

Note: The number of cases and controls for asthma, CAD, and T2D is shown. “/”: we did not consider asthma, CAD, and T2D in the UKB CHI cohort since only very limited cases of these diseases were available.

#### Covariates and selection of significant phenotype-related single-nucleotide polymorphisms for individual-level transPGS

Phenotype-specific covariates used in the UKB cohort are given in the Supplementary File, which either conceptually have an impact on or show a statistically significant connection with analyzed phenotypes. The descriptions of these covariates in different populations are shown in Tables S1–S4. All continuous covariates were standardized. To select phenotype-related SNPs, we downloaded GWAS summary statistics of these phenotypes implemented on individuals of AFR, EAS, or EUR ancestry (Supplementary File and Table S5), and adopted the pruning (*r*^2^ < 0.001 and window size = 1 Mb) and thresholding (*P* < 5 × 10^−8^) method via PLINK [54], with AFR, EAS, or EUR samples in the 1000 Genomes Project as the reference panel. As it has been demonstrated that the PGS constructed via SNPs discovered from diverse populations could improve phenotypic prediction [55], for each phenotype, we thereby calculated the PGS with all SNPs screened from distinct populations no matter whether they were related to that phenotype in the sense of statistical significance.

#### Trans-ancestry genetic similarity and heterogeneity across the EUR and non-EUR populations

Before carrying out genetic prediction with screened SNPs, for each phenotype, we here examined the trans-ethnic genetic similarity and heterogeneity across various populations to demonstrate why we could leverage EUR genetic information in other populations and why we could not apply such information directly in non-EUR populations. The details of used methods are described in the Supplementary File as well as in previous studies [28, 30, 31, 33].

First, to capture an overall picture of common genetic foundation for phenotypes among various populations, we assessed the trans-ethnic genetic overlap under the composite null hypothesis test framework [30] and applied the likelihood ratio test to test the significance of the trans-ethnic genetic overlap proportion (GOP). We also calculated the trans-ethnic genetic correlation (*ρ*) of every phenotype between the non-EUR and EUR populations using popcorn [32].

Second, we conducted a simple linear model for these selected SNPs by regressing the effect sizes of SNPs in the non-EUR population on those in the EUR population [31]. The regression slope offered an insight into *ω* as described in Equation (2), and the *R*^2^ was calculated to assess the prediction capability of SNP effect sizes in the EUR population on the SNP effect sizes in the non-EUR population.

Third, to understand the trans-ethnic genetic heterogeneity of the phenotype across the EUR and non-EUR populations, we examined the difference in heritability (*h*^2^) via an approximate normal test and the difference in linkage disequilibrium (LD) and MAF of selected SNPs with the paired-sample *t*-test [33].

### Cross-validation prediction in UK Biobank and Genetic Epidemiology Research Study on Adult Health and Aging

To quantify the predictive performance of individual-level transPGS, we first carried out an internal Monte-Carlo cross-validation (MCCV) (repeated 50 times) in the UKB and GERA cohorts by calculating *R*^2^ or AUC [41], with 80% individuals as the training data and the rest 20% as the test data.

### Comments regarding real data analysis in summary-level transPGS

These phenotypes were also analyzed by summary-level transPGS (Supplementary File), but additional implementation needed to be pointed out particularly. Following a recent study [31], we obtained multiple SNP sets with various parameters using the P + T method via PLINK and then constructed PGS with SNPs in each set and combined all PGSs via the ridge algorithm by assuming that every PGS had a predictive value on the phenotype. The training data (80% in MCCV) were employed to calculate the weight of each PGS. Because the AFR-ancestry summary statistics of T2D and CAD were unavailable (Table S5), we here did not analyze the two diseases in the GERA AFR population. For further comparison, we also carried out polygenic risk scores-continuous shrinkage (PRS-CS) [56], a novel and widely used summary statistics-based polygenic risk scores (PRS) approach with its strength lying in the continuous shrinkage prior to SNP effect sizes and the utilization of genome-wide markers, which renders it robust across diverse genetic structures and often superior in predictive accuracy compared to other methods. Here, EAS or AFR samples from the 1000 Genomes Project served as the external LD reference panel of PRS-CS.

### Association between polygenic score and the three binary phenotypes

Finally, we assessed the association between PGS and the three diseases in the GERA AFR cohort. To this aim, we calculated PGS via transPGS for individuals of GERA AFR descent using SNP effect sizes estimated from the UKB AFR population. We chose 2.5% as the highest tail and partitioned the PGS into multiple intervals including <2.5%, 2.5% ~ 20%, 20% ~ 80%, 80% ~ 97.5%, and >97.5%, with 20% ~ 80% as the reference to compare individuals to those with average genetic risk. The definition of the highest and lowest 2.5% follows the principle that the reference range for laboratory tests is generally defined by selecting thresholds within which 95% of the individuals fall. Odds ratios (ORs) and 95% confidence intervals (CIs) were reported.

## Results

### Overview of transPGS for genetic prediction in understudied populations

**Figure 1:**
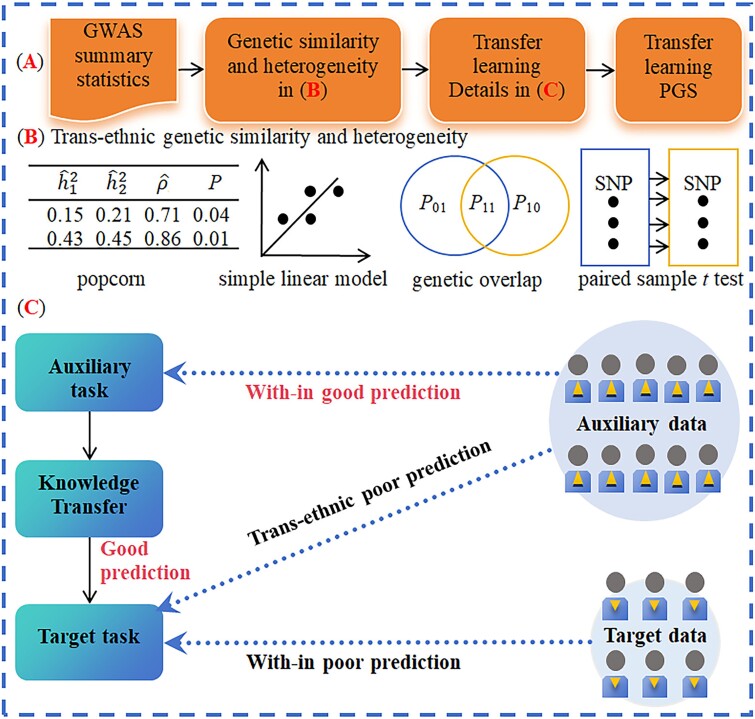
Overview of the proposed transPGS method. (A) General framework to construct transPGS for genetic prediction. (B) Quantifying trans-ethnic genetic similarity and heterogeneity across the EUR and non-EUR populations. (C) Detailed framework of transPGS.

In the machine learning field, transfer learning is recognized as a novel technique that enables the utilization of knowledge acquired from auxiliary samples to enhance learning capability in target samples, which are distinct yet related to the former [37, 43, 57–60]. By borrowing the idea of transfer learning, in this study, we propose transPGS to boost the genetic prediction accuracy in non-EUR populations by leveraging trans-ethnic genetic similarity shared with the EUR population. Theoretically, transPGS helps capture genetic information across diverse ancestral populations and renders the prediction more efficiently and accurately (Fig. 1).

### Results for simulation studies

**Figure 2:**
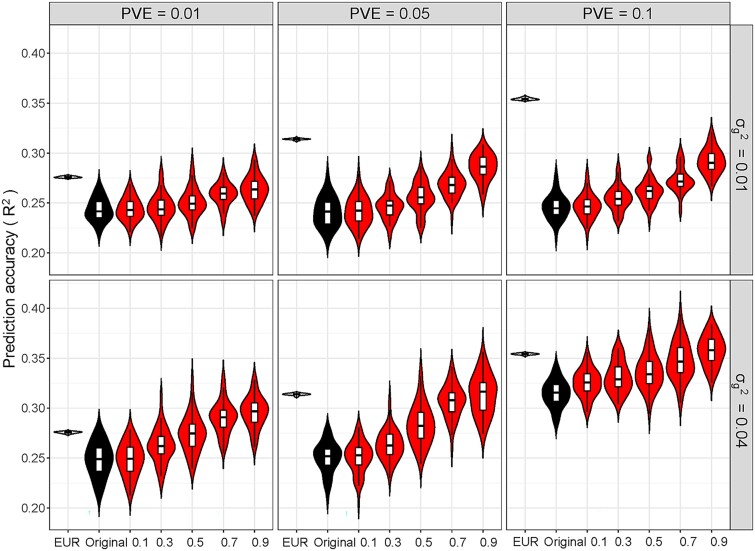
Prediction performance (*R*^2^) of models in the simulations using a set of local SNPs for continuous phenotypes in the target (AFR) and auxiliary (EUR) samples with various degrees of genetic overlap shared between the two populations. *R*^2^ was calculated before and after transfer learning, in which the shared information of the auxiliary population was incorporated into the target population. EUR: PGS model in the auxiliary samples; Original: PGS model in the target samples before transfer learning; *ω* = 0.1, 0.3, 0.5, 0.7, and 0.9 indicates the PGS model in the target samples after transfer learning (i.e. transPGS).

We here mainly reported results observed from individual-level simulations and relegated other results (e.g. those of summary-level simulations) to the Supplementary File. First, the performance with additional auxiliary samples was much better than that obtained with only the target samples (Fig. 2 and Fig. S1), with an average improvement of 26.9% in *R*^2^ for continuous phenotypes and 6.2% in AUC for binary phenotypes across all simulation scenarios.

Second, the prediction accuracy of transPGS was improved with the scale parameter *ω*, which quantified the genetic overlap between the target and auxiliary samples. The correlation between *ω* and the improved prediction accuracy of transPGS was 0.911 for continuous phenotypes and 0.950 for binary phenotypes. For instance, when PVE = 0.05 and ${\boldsymbol{\sigma}}_{\delta}^2$ = 0.04 for continuous phenotypes, the prediction accuracy in the target samples increased by 0.3% for *ω* = 0.1 and 25.6% for *ω* = 0.9 after incorporating auxiliary samples, with an average increase of 12.9% across various values of *ω*. Particularly, when *ω* = 0.9, the prediction performance of transPGS in the target samples after transfer learning almost resembled the PGS constructed directly in the auxiliary samples.

Third, with other parameters unchanged, the prediction performance in both the target and auxiliary samples improved as PVE increased. For instance, when PVE ranged from 0.01 to 0.1 and ${\boldsymbol{\sigma}}_{\delta}^2$ = 0.01, the *R*^2^ in the auxiliary samples elevated from 0.271 to 0.356 and the *R*^2^ in the target samples increased from 0.240 to 0.255 for continuous phenotypes; similar patterns of AUC were observed for binary phenotypes.

Fourth, although the prediction accuracy in the target samples can be enhanced after leveraging the information shared with the auxiliary samples via transfer learning, it was still relatively low compared to that in the auxiliary samples. For instance, when *ω* = 0.9, under which the genetic overlap was the greatest, the prediction accuracy in the target samples can on average achieve 89.7% of that in the auxiliary samples for continuous phenotypes and 98.4% of that in the auxiliary samples for binary phenotypes across all simulation scenarios. Meanwhile, the prediction in the target samples generally presented a greater variation compared to that in the auxiliary samples, which was largely due to the small sample sizes of the target population.

Fifth, similar prediction behaviors were also present for summary-level transPGS (Figs S2 and S3). For instance, when PVE = 0.2 and ${\boldsymbol{\sigma}}_{\delta}^2$ = 0.1, the accuracy of summary-level transPGS increased by 0.9% if *ω* = 0.1 and 27.1% if *ω* = 0.9 for continuous phenotypes after incorporating auxiliary samples, with an average increase of 11.8% across various values of *ω*. Under this simulation setting, an average increase of 5.4% was obtained for binary phenotypes. More simulation results for summary-level transPGS can be found in the Supplementary File.

Finally, the simulation results of transPGS with genome-wide SNPs were largely analogous to those observed using a small set of local SNPs. For individual-level transPGS, its prediction performance was evidently enhanced after incorporating auxiliary samples, leading to an average increase of 13.8% in *R*^2^ for continuous phenotypes and 2.5% in AUC for binary phenotypes across all scenarios (Figs S4 and S5). For summary-level transPGS, the inclusion of auxiliary samples led to an average improvement of 6.1% in *R*^2^ for continuous phenotypes and 1.1% in AUC for binary phenotypes across all scenarios (Figs S6 and S7). Again, the prediction accuracy of transPGS was evaluated as the increase of *ω*.

### Trans-ethnic genetic similarity and genetic heterogeneity

#### Trans-ethnic genetic overlap and genetic correlation

**Figure 3:**
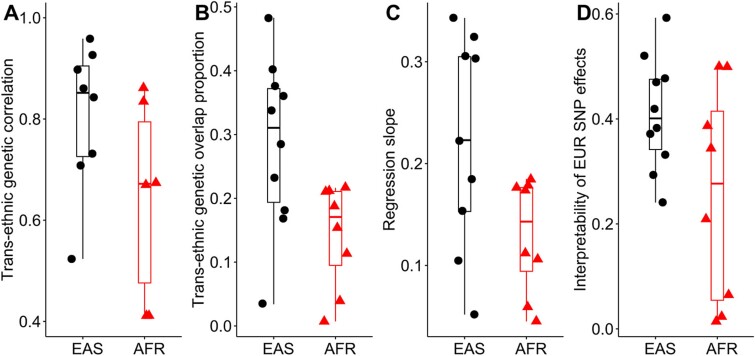
(A) Trans-ethnic genetic correlation between the EAS or AFR and EUR populations. (B) Trans-ethnic genetic overlap proportion between the EAS or AFR and EUR populations. (C) Slope for the phenotype by regressing the SNP effect sizes in the EAS or AFR population on the SNP effect sizes in the EUR population. (D) Estimated *R*^2^ for the phenotype by regressing the SNP effect sizes in the EAS or AFR population on the SNP effect sizes in the EUR population.

As shown in Tables S6 and S7, the trans-ethnic GOPs were evident for all phenotypes in the EAS and EUR populations (from 3.4% for TG to 48.3% for BMI, with an average GOP of 28.6%) as well as in the AFR and EUR populations (from 0.7% for asthma to 21.6% for TG, with an average GOP of 14.2%) if the LD pruning was performed based on EAS-ancestry or AFR-ancestry genotypes (Fig. 3B). Similar genetic overlaps were obtained if the LD pruning was conducted according to EUR-ancestry genotypes.

The substantial genetic overlaps shared between the EUR and non-EUR populations were also supported by high trans-ethnic genetic correlation estimates ($\hat{\rho}$), which ranged from 0.411 ± 0.194 for asthma between the AFR and EUR populations to 0.959 ± 0.069 for TC between the EAS and EUR populations (Fig. 3A and Table S8), and a total of 11 $\hat{\rho}$s did not significantly deviate from one between the AFR and EUR populations or the EAS and EUR populations. The average of $\hat{\rho}$ between the AFR and EUR populations was 0.644, compared to 0.888 between the EAS and EUR populations. Here, the phenotypes with $\hat{\rho}$ larger than one were excluded in the description.

#### Linear regression for single-nucleotide polymorphism effect sizes

The majority of selected SNPs showed consistent genetic effect directions between the AFR and EUR populations (64.1%) (Fig. S8A) as well as between the EAS and EUR populations (71.0%) (Fig. S8B). Further, nearly all the slopes regressing SNP effect sizes in the non-EUR population on those in the EUR population were significantly positive (Table S8), with an average of 0.183 between the EAS and EUR populations and 0.129 between the AFR and EUR populations (Fig. 3C). The *R*^2^ estimates ranged from 0.241 for SBP to 0.593 for LDL with an average of 0.410 in the EAS and EUR populations, and ranged from 0.014 for SBP to 0.500 for HDL with an average of 0.255 in the AFR and EUR populations (Fig. 3D).

### Differences in heritability, genetic correlation, minor allele frequency, and linkage disequilibrium across distinct populations

We observed that some heritability estimates (${\hat{h}}^2$) were significantly different across populations (Table S8). For example, BMI showed the maximal difference in ${\hat{h}}^2$ between the AFR and EUR populations (15.3 ± 1.0% versus 1.5 ± 2.0%, ${P}_{\Delta{\hat{h}}^2}$ = 7.86 × 10^−10^), and DBP had a much larger ${\hat{h}}^2$ in the EAS population than the EUR population (8.6 ± 0.7% versus 3.9 ± 0.4%, ${P}_{\Delta{\hat{h}}^2}$ = 1.97 × 10^−8^). In addition, some trans-ethnic genetic correlation estimates were distinct from one (e.g. asthma and BMI between the AFR and EUR populations), partly reflected by the direction of genetic effect sizes of some SNPs on phenotypes not consistent between the EAS and EUR populations (29.0% on average) or between the AFR and EUR populations (35.9% on average). Moreover, the patterns of MAF and LD between the non-EUR and EUR populations for selected SNPs were substantially different for many analyzed phenotypes (Table S9).

### Results for real data application in the UK Biobank and Genetic Epidemiology Research Study on Adult Health and Aging cohorts

We applied transPGS to the ten phenotypes in the AFR and CHI populations of the UKB cohort and in the AFR and EAS populations of the GERA cohort, with the EUR individuals as auxiliary samples (Tables 2 and 3). Detailed characteristics of these phenotypes are shown in Tables S1–S4.

**Table 2:** Estimated *R*^2^ or AUC of prediction models for the seven continuous phenotypes and three diseases in the UKB CHI population and the GERA EAS population before and after transfer learning with individual-level data.

Phenotype	Original model			Trans-ethnic model		Individual-level transPGS	
	Z (*se*)	Z + G (*se*)	Gain (%)	Z + G (*se*)	Gain (%)	Z + G (*se*)	Gain (%)
UKB (CHI)							
HDL	0.185 (0.025)	0.199 (0.029)	7.57	0.199 (0.029)	7.57	0.245 (0.021)	32.43
LDL	0.024 (0.001)	0.028 (0.001)	16.67	0.029 (0.001)	20.83	0.066 (0.001)	175.00
TC	0.018 (0.007)	0.030 (0.005)	66.67	0.029 (0.004)	61.11	0.047 (0.003)	161.11
TG	0.036 (0.013)	0.043 (0.016)	19.44	0.045 (0.016)	25.00	0.056 (0.017)	55.56
SBP	0.205 (0.021)	0.216 (0.022)	5.37	0.215 (0.021)	4.88	0.251 (0.022)	22.44
DBP	0.117 (0.018)	0.119 (0.017)	1.71	0.119 (0.017)	1.71	0.121 (0.018)	3.42
BMI	0.053 (0.012)	0.058 (0.011)	9.43	0.057 (0.011)	7.55	0.062 (0.011)	16.98
GERA (EAS)							
CAD	0.775 (0.010)	0.790 (0.017)	1.94	0.790 (0.010)	1.94	0.796 (0.017)	2.71
T2D	0.709 (0.008)	0.719 (0.017)	1.41	0.711 (0.008)	0.28	0.727 (0.017)	2.54
Asthma	0.543 (0.019)	0.562 (0.021)	3.50	0.547 (0.019)	0.74	0.565 (0.019)	4.05

Note: Z: covariates only prediction model; original model: prediction model with covariate and PGS; trans-ethnic model: prediction model with covariate and PGS calculated with SNP effect sizes of the EUR population; transPGS: prediction model for the CHI or EAS population after transfer learning of SNP effect sizes by utilizing the genetic information of the EUR population; gain of prediction accuracy was calculated by comparing the PGS model with the covariates only prediction model.

**Table 3:** Estimated *R*^2^ or AUC of prediction models for the seven continuous phenotypes and three diseases in the UKB AFR population and the GERA AFR population before and after transfer learning with individual-level data.

Phenotype	Original model			Trans-ethnic model		Individual-level transPGS	
	Z (*se*)	Z + G (*se*)	Gain (%)	Z + G (*se*)	Gain (%)	Z + G (*se*)	Gain (%)
UKB (AFR)							
HDL	0.166 (0.011)	0.169 (0.011)	1.81	0.112 (0.012)	−32.53	0.192 (0.014)	15.66
LDL	0.051 (0.013)	0.055 (0.015)	7.84	0.065 (0.015)	27.45	0.075 (0.024)	47.06
TC	0.054 (0.012)	0.055 (0.012)	1.85	0.037 (0.015)	−31.48	0.077 (0.021)	42.59
TG	0.094 (0.012)	0.098 (0.011)	4.26	0.066 (0.013)	−29.79	0.116 (0.031)	23.40
SBP	0.162 (0.009)	0.174 (0.012)	7.41	0.164 (0.009)	1.23	0.180 (0.009)	11.11
DBP	0.086 (0.007)	0.088 (0.013)	2.33	0.085 (0.013)	−1.16	0.107 (0.010)	24.42
BMI	0.078 (0.011)	0.084 (0.015)	7.69	0.074 (0.013)	−5.13	0.088 (0.013)	12.82
CAD	0.669 (0.006)	0.689 (0.006)	2.99	0.686 (0.006)	2.54	0.689 (0.006)	2.99
T2D	0.670 (0.004)	0.642 (0.007)	−4.18	0.628 (0.005)	−6.27	0.644 (0.007)	−3.88
Asthma	0.551 (0.005)	0.561 (0.005)	1.81	0.559 (0.005)	1.45	0.562 (0.005)	2.00
GERA (AFR)							
CAD	0.752 (0.009)	0.763 (0.011)	1.46	0.759 (0.009)	0.93	0.763 (0.011)	1.46
T2D	0.605 (0.012)	0.632 (0.012)	4.46	0.624 (0.011)	3.14	0.635 (0.012)	4.96
Asthma	0.584 (0.006)	0.590 (0.023)	1.03	0.596 (0.023)	2.05	0.597 (0.022)	2.23

Note: Z: covariates only prediction model; original model: prediction model with covariate and PGS; trans-ethnic model: prediction model with covariate and PGS calculated with SNP effect sizes of the EUR population; transPGS: prediction model for the AFR population after transfer learning of SNP effect sizes by utilizing the genetic information of the EUR population; gain of prediction accuracy was calculated by comparing the PGS model with the covariates only prediction model.

### Prediction accuracy in the UK Biobank and Genetic Epidemiology Research Study on Adult Health and Aging cohorts

We here described some important findings observed from the real data applications and focused mainly on results obtained from individual-level transPGS. First, compared to the prediction model with only covariates, the accuracy substantially elevated after additionally including genetic risk factors into the AFR or CHI population. For instance, the average prediction gain of the original PGS model was 4.7% in the UKB AFR population and 18.1% in the UKB CHI population across the seven continuous phenotypes. There was an improvement for CAD (3.0%) and asthma (1.8%), but not for T2D (−4.2%) in the UKB AFR population, and increased prediction accuracy was also seen in the GERA EAS population (1.9% for CAD, 1.4% for T2D, and 3.5% for asthma) as well as in the GERA AFR cohort (1.5% for CAD, 4.5% for T2D, and 1.0% for asthma) after considering genetic risk factors.

Second, compared to the original PGS model, the trans-ethnic prediction method, which directly employed the SNP effect sizes estimated from the EUR population to calculate the PGS for individuals of non-EUR descent, often resulted in dissatisfactory performance in the AFR (on average 14.6% decrease) and CHI (on average only 0.4% increase) populations for the seven continuous phenotypes or the three diseases (on average 1.0% decline in the AFR population) in the UKB cohort. On average, 0.3% reduction was observed for the three diseases in the GERA AFR cohort and 1.3% decrease was seen in the GERA EAS cohort.

Third, the accuracy of transPGS was substantially raised after further incorporating the EUR genetic information into the non-EUR population. Very consistent with the higher genetic similarity observed between the EAS and EUR populations than that between the AFR and EUR populations, the improved prediction accuracy for the seven continuous phenotypes was much more pronounced in the UKB CHI population than in the UKB AFR population. For example, compared to the PGS model before transfer learning, the prediction accuracy of transPGS was on average increased by 19.7% in the UKB AFR population and 38.6% in the UKB CHI population across the seven continuous phenotypes but only 0.2% for the three diseases in the UKB AFR population, 0.6% in the GERA AFR population, and 0.8% in the GERA EAS population. Further, very similar results were yielded for individual-level transPGS if using various *P*-value thresholds (e.g. 5 × 10^−6^ and 5 × 10^−7^) to screen SNPs (Tables S10–S13), indicating the robust and superior prediction capability of transPGS.

Fourth, similar to individual-level transPGS, the predictive accuracy of summary-level transPGS was also improved after incorporating EUR genetic information into the CHI or AFR population (Tables 4 and 5). For instance, compared to the original PGS model, the average prediction gains were 7.5% and 14.9% across the continuous phenotypes in the UKB AFR and CHI populations, respectively. However, no significantly increased accuracy was observed for the three diseases; for example, the predictive accuracy was on average improved only by 0.8% for the three diseases in the GERA EAS population and only by 0.2% for asthma in the GERA AFR population.

**Table 4:** Estimated *R*^2^ or AUC of prediction models for the seven continuous phenotypes and three diseases in the UKB CHI population and the GERA EAS population before and after transfer learning with summary-level data.

Phenotype	PGS (*se*)	Summary-level transPGS (*se*)	Gain (%)	PRS-CS (*se*)	Gain (%)
UKB (CHI)					
HDL	0.174 (0.026)	0.204 (0.029)	17.22	0.161 (0.005)	−7.62
LDL	0.016 (0.006)	0.018 (0.009)	13.96	0.011 (0.009)	−29.15
TC	0.023 (0.016)	0.028 (0.017)	25.92	0.023 (0.017)	0.64
TG	0.047 (0.021)	0.050 (0.021)	5.37	0.048 (0.004)	1.96
SBP	0.201 (0.027)	0.234 (0.028)	16.17	0.214 (0.007)	6.63
DBP	0.077 (0.024)	0.084 (0.025)	8.47	0.082 (0.014)	5.87
BMI	0.063 (0.024)	0.074 (0.025)	17.42	0.064 (0.009)	1.72
GERA (EAS)					
CAD	0.779 (0.020)	0.785 (0.021)	0.79	0.778 (0.019)	−0.13
T2D	0.705 (0.019)	0.708 (0.019)	0.52	0.701 (0.007)	−10.08
Asthma	0.535 (0.009)	0.540 (0.009)	1.02	0.514 (0.019)	−27.04

Note: PGS: prediction model before transfer learning; transPGS: prediction model for the CHI or EAS population after transfer learning of SNP effect sizes by utilizing the genetic information of the EUR population; gain of prediction accuracy was calculated by comparing transPGS with PGS before transfer learning.

**Table 5:** Estimated *R*^2^ or AUC of prediction models for the seven continuous phenotypes and one disease in the UKB AFR population and the GERA AFR population before and after transfer learning with summary-level data.

Phenotype	PGS (*se*)	Summary-level transPGS (*se*)	Gain (%)	PRS-CS (*se*)	Gain (%)
UKB (AFR)					
HDL	0.033 (0.009)	0.042 (0.011)	28.58	0.040 (0.016)	22.13
LDL	0.012 (0.007)	0.013 (0.008)	9.39	0.012 (0.006)	0.48
TC	0.019 (0.010)	0.020 (0.010)	7.36	0.017 (0.012)	−10.35
TG	0.029 (0.008)	0.030 (0.008)	2.36	0.029 (0.001)	−1.09
SBP	0.149 (0.015)	0.155 (0.013)	4.13	0.155 (0.009)	3.53
DBP	0.033 (0.007)	0.033 (0.007)	1.15	0.033 (0.018)	−0.43
BMI	0.012 (0.006)	0.012 (0.006)	0.03	0.006 (0.015)	−52.82
GERA (AFR)					
Asthma	0.528 (0.027)	0.529 (0.027)	0.18	0.528 (0.008)	0.10

Note: PGS: prediction model before transfer learning; transPGS: prediction model for the AFR population after transfer learning of SNP effect sizes by utilizing the genetic information of the EUR population; gain of prediction accuracy was calculated by comparing transPGS with PGS before transfer learning.

Fifth, largely consistent with the simulations, the accuracy gain of transPGS was positively related to the slope of regressing SNP effect sizes in the non-EUR population on those in the EUR population (*r* = 0.658 and *P* = .002 for individual-level transPGS, *r* = 0.631 and *P* = .005 for summary-level transPGS).

Sixth, as a negative control, rather than using the EUR population as auxiliary samples, we performed similar transPGS analyses in the UKB or GERA cohort by attempting to incorporate genetic formation of the AFR population into the EAS (or CHI) population or vice versa. Interestingly, we found that transPGS behaved considerably poorly under this setting (Tables S14–S17). It was partly due to the much smaller AFR (EAS or CHI) samples relative to the larger EUR samples (e.g. on average, 30-fold larger for summary-level transPGS; see Table S5), which implied that no sufficient information could be integrated from the auxiliary population into the target population.

Finally, compared to the PGS model before transfer learning, PRS-CS behaved better for some of these phenotypes or diseases but displayed consistently less satisfactory predictive ability compared to transPGS.

### Association between polygenic score and three binary phenotypes

**Figure 4:**
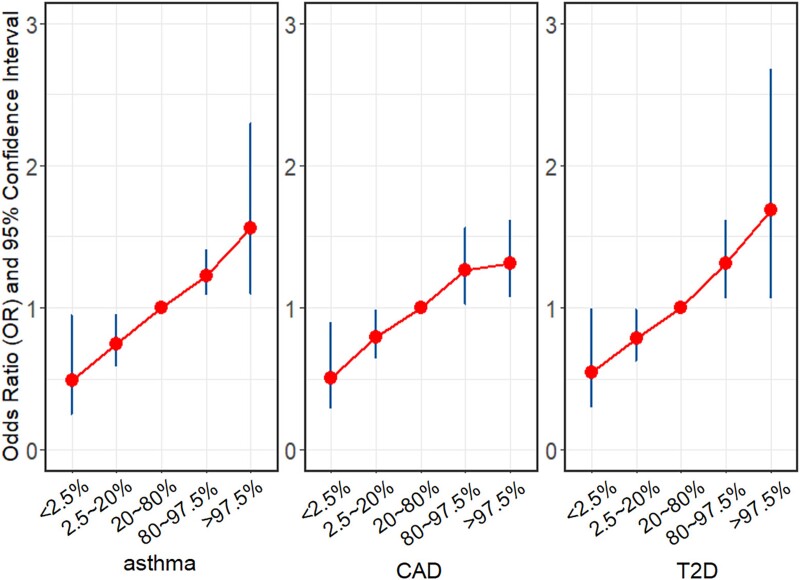
Risk of occurring CAD, T2D, and asthma in the GERA AFR cohort for participants with the genetic risk being in various intervals of the PGS distribution. The PGS was calculated by individual-level transPGS; the 20% ~ 80% PGS was used as the reference.

In the association analysis of the GERA AFR cohort, according to individual-level transPGS, we observed an increasing trend that higher PGS often led to greater risk for all three diseases (Fig. 4). Specifically, compared to average PGS (20 ~ 80th percentile of the PGS distribution), being in the top 2.5% of the distribution translated into significantly greater OR, ranging from 1.56 (1.10 ~ 2.30) for asthma to 1.31 (1.07 ~ 1.61) for CAD and 1.69 (1.06 ~ 2.67) for T2D. Similarly, when comparing average PGS to the lowest 2.5%, the OR evidently reduced, ranging from 0.49 (0.25 ~ 0.95) for asthma to 0.51 (0.29 ~ 0.89) for CAD and 0.54 (0.30 ~ 0.99) for T2D. Similar association patterns were also observed for the PGS calculated with summary-level transPGS (Fig. S9).

## Discussion

### Summary of our work

In this study, we have developed a novel method called transPGS for genetic prediction in non-EUR populations by integrating shared genetic information from the EUR population. Through simulations and real data applications to 10 phenotypes available from the EAS and AFR populations with small sample sizes and from the EUR population with large sample sizes, we demonstrated the usefulness and robustness of transPGS in elevating prediction accuracy among underrepresented populations. Our work highlights the urgent need to substantially increase participant diversity and boost the portability of PGS in genetic studies, especially from the most underrepresented regions such as AFR, to avoid exacerbation of health disparities in the era of precision medicine and precision public health.

### Genetic similarity and heterogeneity

To show the solid foundation for transPGS in our real data applications, we carried out a comprehensive genetic similarity analysis and found significant genetic similarity between the EUR and EAS populations as well as between the EUR and AFR populations. We observed that nearly all the trans-ethnic genetic correlations were larger than zero and screened SNPs generally exhibited consistent effect directions across various populations. These high consistencies implied that non-EUR individuals can benefit from the genomic research implemented in those of EUR ancestry. Therefore, we can reasonably utilize EUR information to improve the prediction ability of non-EUR models, which forms the biological basis underlying our study to conduct transPGS [28, 30–32].

On the other hand, we also discovered that even population-common SNPs showed a varying degree of heterogeneity in genetic architecture between the non-EUR and EUR populations. Recent theoretical and empirical studies have demonstrated that information gaps exist across diverse populations, especially when the EUR and non-EUR samples are genetically distant from each other [61, 62]. As detected in our study, this is possible due to distinctions in genetic architecture (such as MAF and LD) and environmental exposures of diverse populations [9, 62]. For instance, much longer blocks of LD exist in the EUR population compared to the AFR population [19] and MAFs often vary widely between the EAS and EUR populations [63]. Therefore, associated SNPs discovered in the EUR population cannot be completely and directly transferred to other populations. These genetic discrepancies across ancestry groups offer a critical interpretation for the poor portability of PGS prediction across distinct populations, where the direct use of PGS models trained with EUR data to non-EUR individuals often leads to reduced prediction accuracy.

### Various performance of transPGS in the EAS and AFR populations

Very importantly, transPGS exhibited better learning performance in the EAS population compared to the AFR population because the former was genetically close to the EUR population compared to the latter [64]. For instance, in the genetic similarity analysis where the SNP effect sizes of the EAS or AFR population were regressed against those of the EUR population, the interpretability of the SNP effect sizes in the EAS population surpassed that of the AFR population, as reflected by the higher average *R*^2^ (0.319 versus 0.246).

In the genetic heterogeneity analysis, we observed that the difference in LD between the EUR and AFR populations was more pronounced than that between the EUR and EAS populations. Thereby, we can expect that the predictive accuracy by transferring EUR genetic data into non-EUR ancestries would be improved notably higher in the EAS population than in the AFR population. Previous studies also demonstrated these observations [65, 66].

### Bayesian perspective of transPGS

Methodologically, transPGS can be explained from a Bayesian perspective [67], where the SNP effect sizes of the EUR population are integrated into understudied populations through a prior function. The widely observed trans-ethnic genetic similarity across distinct populations suggests that this prior is informative and can lead to more accurate genetic prediction. Instead of using sampling techniques or variational methods as done in the classical Bayesian model [41], we here fit transPGS in a probabilistic way. Additionally, because hundreds of thousands of samples are often analyzed in the EUR population (the standard error of genetic effect is thus rather small), for simplicity individual-level transPGS ignores the uncertainty of SNP effect sizes in the auxiliary population when transfer learning genetic information from the EUR population to non-EUR populations. However, we implicitly consider the auxiliary uncertainty of SNP effect sizes in summary-level transPGS by re-estimating marginal EUR effects of the auxiliary population through ridge regression. Furthermore, the model framework of transPGS is similar to the two-step estimation presented in transfer learning of prediction with proxy data [37], where it has been proven that the two-step estimators are optimal in the sense of lower parameter estimation error.

### Comparison to other polygenic score methods and strengths of transPGS

Although a range of trans-ethnic PGS models have been recently developed [34], transPGS possesses its own advantages. First, compared to MutiPRS [68] and PolyPred [69], which fail to utilize trans-ethnic genetic similarity [70], transPGS can more effectively integrate multi-ethnic genetic similarity. Second, compared to XP-BLUP [71] and CT-SLEB [31], which are also established based on the traditional P + T method, transPGS further explains LD structures that are typically distinct across various ancestral groups.

Third, some previous trans-ethnic PGS methods, such as SDPRX, BridgePRS, PRS-CSx [70], and X-Wing [72], are constructed under the Bayesian framework and fitted via sampling algorithms and thus are computationally slow and generally demand more computational resources, making them difficult to apply to GWAS summary statistics with high-density SNPs. In contrast, the computational speed of transPGS is much faster as only screened SNPs are included. Fourth, some trans-ethnic PGS methods such as SDPRX [73], XPXP [74], and XPASS [75] can only be applied to trans-ethnic prediction with only two populations or cannot further incorporate other useful genetic annotations such as expression quantitative trait loci (eQTL) and protein quantitative trait loci (pQTL), transPGS can be employed to two or more auxiliary populations and simultaneously incorporate other genetic information using a conventional manner via the regression framework [76, 77].

Particularly, we discovered that transPGS, through explicitly leveraging informative external knowledge, also demonstrated higher prediction accuracy compared to PRS-CS [56], which utilized genome-wide SNPs and outperformed the PGS model before transfer learning. This may be due to smaller sample sizes of underrepresented populations, leading to less precise and unstable effect size estimates. Meanwhile, it also implies that some well-known conclusions, such as genome-wide SNPs resulting in better prediction capability, may not hold when only a few samples can be available, and that there likely exists a trade-off between more SNPs and smaller samples for more accurate genetic prediction in understudied populations.

### Limitations of our study

Despite these strengths mentioned above, our work has limitations and leaves several questions needing further exploration. First, transPGS showed little or no improvement for diseases, suggesting that PGS may have a weak impact at the population level and a limited role in improving risk prediction of some specific diseases, consistent with some prior findings [18, 78, 79].

Second, although we demonstrated large relative improvements in prediction accuracy by transfer learning in understudied populations, the absolute gain of transPGS was still not high enough to achieve accurate clinical utility for these analyzed phenotypes, especially diseases [80]. Third, transPGS was limited to common variants, but it is hoped that the influence of rare variants (e.g. MAF < 0.01) could be included in future work [81–84]. Meanwhile, the impact of the X chromosome, whose information may also contribute to the accuracy of prediction [85], was not yet considered at present.

Fourth, the parameter estimation of transPGS is not computationally efficient because it involves the operation of a square matrix with its dimension equal to the number of included SNPs. Therefore, the computation of transPGS becomes difficult when a large number of SNPs are incorporated, limiting its application to genome-wide genetic variants. Instead, we have to carefully select SNPs when performing transPGS, although this strategy is not uncommonly employed in many applied fields and statistical genetics because it is computationally easier and, sometimes, only summary information of significant SNPs can be publicly available. Nevertheless, in the future, we expect to expand transPGS to the application of genome-wide SNPs while guaranteeing computational feasibility in two aspects. First, one can threshold and select SNPs under various conditions, create a PRS for each condition, and finally combine them into a single PRS (sometimes also select an optimal one with the highest prediction accuracy but at the risk of over-fitting), as done in our summary-level transPGS. Second, one can perform transPGS across the whole genome in a sliding window manner and then integrate the generated PRSs of all windows into a single score. However, both strategies need additional phenotype and genotype datasets to produce weights for pooling PRSs under distinct conditions or windows. In practice, there may be not sufficient samples for accurate weight estimation, especially for genetic prediction in small samples of underrepresented populations.

## Conclusions

The proposed transPGS represents a flexible and effective PGS method, which offers more accurate genetic prediction in understudied populations.

**Key Points**

- A new and computationally scalable polygenic score method, transPGS, has been proposed, which provides a flexible, accurate, and effective method for genetic prediction in underserved populations.
- The usefulness of transPGS in elevated prediction accuracy has been extensively demonstrated through individual-level and summary-level simulations as well as the successful application to ten traits.
- We further reveal distinct LD and MAF patterns across ancestral groups contribute the dissatisfactory portability of PGS.

## Supplementary Material

2025-01-09_transPGS_SupplementaryFile_bbaf048

## Data Availability

Researchers can access the UK Biobank data by applying to the UK Biobank official website (https://www.ukbiobank.ac.uk/) and the GERA data by applying to the dbGaP. All data generated or analyzed during this study are included in this published article and its supplementary data.
