# Supplementary material for: Polygenic prediction for underrepresented populations through transfer learning by utilizing genetic similarity shared with European populations
Source: Brief Bioinform. 2025 Feb 5;26(1):bbaf048. doi: 10.1093/bib/bbaf048 (PMC11794457; doi:10.1093/bib/bbaf048)
Supplement: 2025-01-09_transPGS_SupplementaryFile_bbaf048 [file 2025-01-09_transpgs_supplementaryfile_bbaf048.docx]

# Supplementary File

# Summary-level transPGS

## Distribution of summary statistics

Let $\hat{\beta}$=($\hat{\beta}_{1}$, …, $\hat{\beta}_{P}$) be the vector of marginal effect sizes of *P* particularly selected single-nucleotide polymorphisms (SNPs) in the target population, $\hat{\mathbf{S}}$ be a diagonal matrix with its element the standard error of marginal effect sizes (i.e., ${\hat{\mathbf{S}}}_{pp}=se(\hat{\beta}_{p})$, *p*=1, …, *P*). Within the context of polygenic architecture of complex phenotype [[1](#_ENREF_1)], we assume the following relation

$$\hat{\beta} \text{\textasciitilde} \text{MVN}(\hat{S}R\hat{S}^{-1}\beta, \text{σ}_{e}^{2} \times\hat{S}R\hat{S})$$

where MVN(***μ***, **Σ**) denotes the multivariate normal distribution with the mean vector of ***μ*** and the variance matrix of **Σ**; **R** is the linkage disequilibrium (LD) matrix; ***β***=($\beta_{1}$, …, $\beta_{P}$) is the vector of joint SNP effect sizes on the phenotype in the target population; $\sigma_{e}^{2}$ is an additional variance explaining potential discrepancies such as measurement error when calculating LD from external reference panels (i.e., difference of LD estimated in-sample and out-sample) and potential information loss when using summary-level data rather than individual-level data. When $\sigma_{e}^{2}$=1 it exactly reduces to the regression model of summary statistics given in [[2](#_ENREF_2)].

Because of unavailability of individual-level genotypes, **R** is calculated with genotypes of population-matched individuals from external reference panels such as the 1000 Genomes Project [[3](#_ENREF_3)]. Although many sophisticated approaches have been proposed [[4](#_ENREF_4)], we calculate **R** in a shrinkage, computationally simple manner

$$R \text{=} \kappa\times\hat{R} \text{+} (1 - \kappa\text{)} \times I_{P}$$

where $\hat{\mathbf{R}}$ is the empirical LD matrix estimated from the reference genotypes, *κ* is the shrinkage parameter and **I***_P_* is a *P*-dimensional identify matrix. We specify *κ*=0.95 throughout our analyses in terms of the previous recommendation [[5](#_ENREF_5)].

## Regression likelihood for summary statistics

To estimate ***β*** in model , we obtain the log-likelihood by ignoring the constant term

$$LogL(\beta|\hat{\beta}) = -\frac{1}{2}(\hat{\beta} - \hat{S}R\hat{S}^{-1}\beta)^{T}(\text{σ}_{e}^{2}\hat{S}R\hat{S})^{-1}(\hat{\beta} - \hat{S}R\hat{S}^{-1}\beta)$$

It is easy to see that Equation is actually the log-likelihood of a linear model with $\hat{\boldsymbol{\beta}}$ regressing on $\hat{\mathbf{S}}\mathbf{R}{\hat{\mathbf{S}}}^{-1}$weighted by $\hat{\mathbf{S}}\mathbf{R}\hat{\mathbf{S}}$. Thus, we have an equivalent relation

$$Y' = W\beta+ e, e \text{\textasciitilde} \text{MVN(0,} \text{σ}_{e}^{2}I_{P}\text{)}$$

where $\boldsymbol{Y}^{'}={(\hat{\mathbf{S}}\mathbf{R}\hat{\mathbf{S}})}^{-\frac{1}{2}}\hat{\boldsymbol{\beta}}$, $\mathbf{W}={(\hat{\mathbf{S}}\mathbf{R}\hat{\mathbf{S}})}^{-\frac{1}{2}}\hat{\mathbf{S}}\mathbf{R}{\hat{\mathbf{S}}}^{-1}$, and ***e*** represents the residuals.

In addition to target samples, suppose that we also observe additional samples from the auxiliary population, which is informative and provides substantial assistance to the target model. Similar to the principle for the target population, we obtain

$$Y_{t}^{'} = W_{t}b_{t} + e_{t}, e_{t}\text{\textasciitilde} \text{MVN(0,} \text{σ}_{e_{t}}^{2}I_{P}\text{)}$$

where $\boldsymbol{Y}_{t}^{\boldsymbol{'}}$, $\mathbf{W}_{t}$, $\boldsymbol{e}_{t}$ and $\sigma_{e_{t}}^{2}$ have the identical definitions as those in the target model, and $\boldsymbol{b}_{t}$ denotes the joint SNP effect sizes in the auxiliary model. Note that, both models and have *P* samples and *P* unknown parameters. For simplicity and stability, we follow the assumption of polygenicity and employ the ridge regression algorithm for parameter estimation in the two models [[6](#_ENREF_6)]. By giving up the unbiasedness of the least squares method, ridge model can more stably generate regression coefficients (i.e., genetic effects in our applied context) even under ill-conditioned situations. Compared to other penalization methods such as Lasso [[7](#_ENREF_7), [8](#_ENREF_8)], ridge regression usually does not shrink all regression coefficients exactly to zero; therefore, SNPs can be retained in the prediction model, which is more in line with the actual situation that many phenotypes are polygenetic [[1](#_ENREF_1)]. We implement ridge regression via the R glmnet package [[9](#_ENREF_9)], with the tune parameter selected through built-in cross-validation.

## Integrate auxiliary information into the target samples

Again, since $\boldsymbol{b}_{t}$ and ***β*** are obtained from different samples, they are not identical. To effectively transfer existing genetic knowledge from the auxiliary samples to the target samples, we characterize the relation between these two sets of effect sizes by treating ***β*** as random effects in a prior function

$$\beta\text{=} \sum_{t = 1}^{T} b_{t}\omega_{t} + \delta, \delta\text{\textasciitilde} \text{MVN(0,} \text{σ}_{g}^{2}I_{P}\text{)}$$

where *T* is the total number of auxiliary populations, *ω_t_* is a scale parameter, an informative auxiliary study implies that *ω_t_* is different from zero; and ***δ***=(*δ*_1_, …, *δ_P_*) is the vector of target-specific SNP effect sizes following a normal distribution (e.g., *δ_p_*~*N*(0,$\sigma_{\delta}^{2}$), *p*=1, …, *P*), which can be referred to as a discrepancy vector quantifying the similarity of effect sizes between the target and auxiliary samples [[10](#_ENREF_10), [11](#_ENREF_11)].

Then, plugging into , we have

$$Y' \text{=} W\sum_{t = 1}^{T} \omega_{t}b_{t} + W\delta+ e, e \text{\textasciitilde} \text{MVN(0,} \text{σ}_{e}^{2}I_{P}\text{)}$$

To enhance the computational speed of parameter estimation, we here apply the parameter expansion expectation maximization (PX-EM) algorithm given below [[11-15](#_ENREF_11)] to estimate unknown parameters in model .

# Parameter estimation algorithms in transPGS

## PX-EM: parameter expansion expectation maximization for transPGS under the framework of linear mixed model for genetic prediction of continuous phenotypes

The PX-EM method is a covariance-adjusted EM algorithm through efficient expanded data augmentation [[11-15](#_ENREF_11)], and can be easily implemented by introducing an extra multiplicative expansion parameter (say γ) for random effects to accelerate the convergence of the conventional EM algorithm, which is key for the setting where the variance is on the boundary of parameter space.

For individual-level transPGS with a continuous phenotype, the expanded mixed-effects form can be simply expressed as [[11](#_ENREF_11), [15](#_ENREF_15)]

$$Y \text{=} X\alpha+ Z\omega+\text{γ}G\delta+ e$$

where ***Y*** is an *n×*1 vector of the continuous phenotype (e.g., lipids), with *n* the sample size; **X** is an *n×m* matrix of conventional covariates with the first column consisting of ones for intercept, with *m* the number of covariates; ***α*** is the fixed effect vector for covariates; **Z***=*[**G*b***_1_, …, **G*b****_T_*] is an *n×T* matrix of trans-ethnic genetic risk scores weighted by effects ***b****_t_* available from auxiliary populations, with *T* the total number of auxiliary populations; and ***ω*** is a *T×*1 effect vector of trans-ethnic genetic risk scores; **G** is an *n×P* matrix of genotypes for a group of previously selected SNPs in the target population, with *P* the number of SNPs; ***δ*** is the random effect vector for selected SNPs and assumed to follow a normal distribution (i.e., *δ_j_*~*N*(0,$\sigma_{\delta}^{2}$), *j*=1, …, *P*) since a large number of variants are involved [[16](#_ENREF_16)]; and ***e*** is the vector of normally distributed residual errors (i.e., *e_i_*~*N*(0,$\sigma_{e}^{2}$), *i*=1, …, *n*). As seen, model has the similar form to model , thus can be also easily estimated with PX-EM.

Let$\mathbf{E}$***=***(X, **Z**) and **λ*=***(***α***, ***ω***); we treat ***δ*** to be the latent variable and let ***θ***=(**λ**, ***δ***, γ,$\sigma_{\delta}^{2}$, $\sigma_{e}^{2}$) be all unknown parameters in model . The complete data log-likelihood is

$$\begin{matrix} LogL\text{(}\theta\text{)} = -\frac{1}{2\text{σ}_{e}^{2}}(Y - E\lambda- \text{γ}G\delta)^{T}(Y - E\lambda- \text{γ}G\delta) \\ -\frac{1}{2}n\log(2\text{πσ}_{e}^{2}) - \frac{1}{2\text{σ}_{\delta}^{2}}\delta^{T}\delta- \frac{1}{2}P\log(2\text{πσ}_{\delta}^{2}) \end{matrix}$$

To conduct the PX-EM estimation, we first obtain the expectation of ***δ*** with regards to all other parameters. Ignoring the constant in the log-likelihood, the terms including ***δ*** are

$$-\frac{1}{2}\delta^{T}(\frac{1}{\text{σ}_{e}^{2}}\text{γ}^{2}G^{T}G + \frac{1}{\text{σ}_{\delta}^{2}}I_{P})\delta+ \frac{1}{\text{σ}_{\delta}^{2}}\text{γ}(Y - E\lambda)^{T}G\delta$$

As it shows a quadratic form; the posterior distribution of ***δ*** is a multivariate normal distribution with the mean vector and variance matrix as

$$\begin{matrix} \mu_{\delta} = \frac{1}{\text{σ}_{e}^{2}}\text{γ}(\frac{1}{\text{σ}_{e}^{2}}\text{γ}^{2}G^{T}G + \frac{1}{\text{σ}_{\delta}^{2}}I_{P})^{-1}G^{T}(Y - E\lambda) \\ \Sigma_{\delta} = (\frac{1}{\text{σ}_{e}^{2}}\text{γ}^{2}G^{T}G + \frac{1}{\text{σ}_{\delta}^{2}}I_{P})^{-1} \end{matrix}$$

***E-step***

In the E-step, we derive the *Q* function through taking the expectation of the complete data log-likelihood with respect to ***δ***. We have E(***δ****^T^***M*δ***) = ***μ****^T^***M*μ*** + *tr*(**MΣ**) for any symmetric matrix **M** if ***δ*** is normally distributed with mean ***μ*** and variance **Σ** (here *tr*(**M**) denotes the trace of a square matrix **M**). Let ***r***=***Y***-**Eλ**-γ**Gδ**; for the terms involving ***δ*** in the log-likelihood function in , we have

$$\begin{matrix} \text{E(}r^{T}r\text{)} = \text{E}(r^{T}r) = \text{E}\{(Y - E\lambda- \text{γ}G\delta)^{T}(Y - E\lambda- \text{γ}G\delta)\} \\ = \text{E}\left\{(Y - E\lambda)^{T}(Y - E\lambda)\right. - 2\text{γ}(Y - E\lambda)^{T}G\delta\left. \text{+} \text{γ}^{2}\delta^{T}G^{T}G\delta)\right\} \\ = (Y - E\lambda)^{T}(Y - E\lambda) - 2\text{γ}(Y - E\lambda)^{T}G\mu_{\delta} \\ \text{+} \text{γ}^{2}\mu_{\delta}^{T}G^{T}G\mu_{\delta} \text{+} \text{γ}^{2}tr(G^{T}G\Sigma_{\delta}) \\ \text{E}(\delta^{T}\delta) = \mu_{\delta}^{T}\mu_{\delta} \text{+} tr(\Sigma_{\delta}) \end{matrix}$$

Then, given the current estimates of ***θ***^(^*^t^*^-1)^, the *Q* function is

$$\begin{matrix} Q\text{(}\theta^{(t)}\text{|}\theta^{(t-1)}\text{)} \text{=}- \frac{1}{2\sigma_{e}^{2}}(Y - E\lambda- \text{γ}G\mu_{\delta})^{T}(Y - E\lambda- \text{γ}G\mu_{\delta}) \\ -\frac{1}{2}n\log(2\text{π}\sigma_{e}^{2}) -\frac{1}{2}P\log(2\text{π}\sigma_{\delta}^{2}) - \frac{1}{2\sigma_{\delta}^{2}}\mu_{\delta}^{T}\mu_{\delta} \\ -\frac{1}{2}tr\left\{(\frac{1}{\sigma_{e}^{2}}\text{γ}^{2}G^{T}G + \frac{1}{\sigma_{\delta}^{2}}I_{P})\Sigma_{\delta}\right\} \end{matrix}$$

***M-step***

In the M-step, we obtain the new update for ***θ*** (i.e., ***θ***^(^*^t^*^)^) by setting the first derivative of *Q* function to zero for each of the unknown parameters. We have

$$\begin{matrix} \lambda\text{=} (E^{T}E)^{-1}E^{T}(Y - \text{γ}G\mu_{\delta}) \\ \sigma_{e}^{2} \text{=} \frac{1}{n}\left\{(Y - E\lambda- \text{γ}G\mu_{\delta})^{T}(Y - E\lambda- \text{γ}G\mu_{\delta}) + tr(\text{γ}^{2}G^{T}G\Sigma_{\delta})\right\} \\ \sigma_{\delta}^{2} \text{=} \frac{1}{P}\left\{\mu_{\delta}^{T}\mu_{\delta} + tr(\Sigma_{\delta})\right\} \\ \text{γ} \text{=} \left\{\mu_{\delta}^{T}G^{T}G\mu_{\delta} \text{+} tr(G^{T}G\Sigma_{\delta})\right\}^{-1}(Y - E\lambda)^{T}G\mu_{\delta} \end{matrix}$$

Finally, we summarize the PX-EM algorithm below. Although PX-EM needs an extra computation for the expansion parameter γ (i.e., the last line in ) in each update iteration, the cost is ignorable compared to the slow convergence of the standard EM algorithm [[12](#_ENREF_12)]. Fixing γ=1 in the PX-EM algorithm leads to the conventional EM algorithm. It has been proven that the rate of PX-EM is at least as quick as the general EM since it implements a more efficient update iteration in the M-step for the expanded model [[17](#_ENREF_17)]. Simulation studies have also shown that the PX-EM algorithm can substantially improve the convergence [[15](#_ENREF_15)].

PX-EM algorithm for individual-level transPGS with continuous phenotypes

| step 1. | set initial value for ***θ***, say ***θ***^(0)^, and set *t* = 1; |
| --- | --- |
| step 2. | compute $\mu_{\delta}$ and $\Sigma_{\delta}$ in the E-step in and evaluate the *Q* function in ; |
| step 3. | update the parameters given in the M-step in and obtain ***θ***^(^*^t^* ^+ 1)^; |
| step 4. | perform a reduction step by rescaling $\sigma_{\delta}^{(t + 1)} = (\gamma^{(t + 1)})^{2}\sigma_{\delta}^{(t + 1)}$ and setting $\text{γ}^{(t + 1)}$=1; |
| step 5. | set *t* = *t* + 1; repeat step 2 and step 4 until convergence to obtain the final estimates. |

## AI-REML: average information restricted maximum likelihood for transPGS under the framework of generalized linear mixed model estimated with pseudo quasi-likelihood for genetic prediction of binary phenotypes

Using the same notations as given for transPGS with a binary phenotype, we have the following logistic mixed model

$$\eta= g(\mu) = X\alpha+ Z\omega+G\delta= E\lambda+G\delta= E\lambda+u, u \sim N(0, \text{σ}_{\delta}^{2}K)$$

where *g* denotes the logit link function, ***Y*** is an *n×*1 vector of the binary phenotype such as disease status, and **K**=**GG***^T^*. The AI-REML algorithm is used to fit this logistic mixed models [[18-23](#_ENREF_18)] and performs parameter estimation through the pseudo quasi-likelihood (PQL) [[23](#_ENREF_23), [24](#_ENREF_24)]. To this aim, we first define the pseudo-data (also called working response variable)

$$\tilde{Y}=\eta+g'(\mu)(Y-\mu)$$

Under the condition of known $\sigma_{\delta}^{2}$, we obtain

$$\hat{\lambda} = (E^{T}\mathbf{H}^{-1}E)^{-1}E^{T}H^{-1}\tilde{Y} \text{and} \hat{u} = \mathbf{VH}^{-1}(\tilde{Y}-E\hat{\lambda})$$

where **H**=**D**^-1^+**V**, with the diagonal matrix **D**=$\mathrm{diag}\left( 1/{g'\left( \mu_{i} \right)} \right)$ and **V**=$\sigma_{\delta}^{2}$**K**+**I***_n_*.

Next, we estimate $\sigma_{\delta}^{2}$ conditional on the current estimates of $\boldsymbol{u}$ and **λ**. To this goal, we integrate out **λ** to construct the following restricted maximum likelihood after ignoring the constant term

$$LogL_{R}(\text{σ}_{\delta}^{2})=-\frac{1}{2}\left(\text{log}|\mathbf{H}|+\text{log}|E^{T}\mathbf{H}^{-1}E|+\tilde{Y}^{T}\mathbf{P}\tilde{Y}\right)$$

where $\mathbf{P}= \mathbf{H}^{-1}-\mathbf{H}^{-1}\mathbf{E}^{T}\left( \mathbf{E}^{T}\mathbf{H}^{-1}\mathbf{E} \right)^{-1}\mathbf{E}\mathbf{H}^{-1}$. Then, we yield the first derivative regarding $\sigma_{\delta}^{2}$

$$U=\frac{\partial LogL_{R}(\text{σ}_{\delta}^{2})}{\partial\text{σ}_{\delta}^{2}}=\frac{1}{2}\left\{\tilde{Y}^{T}\mathbf{PKP}\tilde{Y}-tr(\mathbf{PK})\right\}$$

as well as the second derivative

$$\frac{\partial^{2}LogL_{R}(\text{σ}_{\delta}^{2})}{\partial\text{σ}_{\delta}^{2}\partial\text{σ}_{\delta}^{2}} = \frac{1}{2}tr(\mathbf{KPKP})-\tilde{Y}^{T}\mathbf{PKPKP}\tilde{Y}$$

The second derivative constitutes the observed information matrix. For a symmetric matrix **A** [[25](#_ENREF_25)], we can yield the expectation of the second derivative

$$E\left[\frac{\partial^{2}LogL_{R}(\text{σ}_{\delta}^{2})}{\partial\text{σ}_{\delta}^{2}\partial\text{σ}_{\delta}^{2}}\right] = -\frac{1}{2}tr(\mathbf{KPKP})$$

Furthermore, following previous studies [[18-23](#_ENREF_18)], we define the average information matrix (AI) as the average of the observed information matrix and expected information matrix

$$\mathbf{AI}=\tilde{Y}^{T}\mathbf{PKPKP}\tilde{Y}$$

With the first and second order derivatives, we perform Newton-Raphson update with the AI-REML algorithm and obtain the estimate of $\sigma_{\delta}^{2}$

which in turn leads to estimates of $\boldsymbol{u}$ and **λ**.

Finally, we summarize the AI-REML algorithm below, which estimates unknown parameters in individual-level transPGS with binary phenotypes.

AI-REML algorithm for individual-level transPGS with binary phenotypes

| step 1. | Give $\boldsymbol{\lambda}^{\left( 0 \right)}$ and ${\sigma_{\delta}^{2}}^{\left( 0 \right)},$ generate the pseudo-data ${\tilde{\boldsymbol{Y}}}^{\left( 0 \right)}$; and set $t=1$; |
| --- | --- |
| step 2. | Update ${\sigma_{\delta}^{2}}^{\left( t \right)}$ according to ; |
| step 3. | Update $\boldsymbol{\lambda}^{\left( t \right)}$ and $\boldsymbol{u}^{\left( t \right)}$ in terms of with ${\sigma_{\delta}^{2}}^{\left( t \right)}$ and ${\tilde{\boldsymbol{Y}}}^{\left( t-1 \right)}$; |
| step 4. | Update ${\tilde{\boldsymbol{Y}}}^{\left( t \right)}$ according to using $\boldsymbol{\lambda}^{\left( t \right)}$ and $\boldsymbol{u}^{\left( t \right)}$; |
| step 5. | set *t* = *t* + 1; repeat step 2 and step 4 until convergence to obtain the final estimates. |

# Simulation studies for summary-level transPGS

Similar to individual-level transPGS, extensive simulations were also conducted to evaluate the prediction performance of summary-level transPGS. We generated continuous or binary phenotypes with genotypes available from the UK Biobank (UKB) cohort [[26](#_ENREF_26)]. To mimic the real data applications below, we randomly selected 130,000 individuals of European (EUR) descent, and created the continuous phenotype in the auxiliary population via a general linear model with 1,500 common SNPs and two covariates (a binary one and a continuous one). These SNPs were obtained from a continuous local region of Chr 1 and had minor allele frequency (MAF) >1%. The effect size of each covariate was set to 0.5, and the effect sizes (***b***) of SNPs were sampled from a normal distribution with a mean of zero and a specific variance so that the phenotypic variance explained (PVE) by SNPs was 10%, 20%, or 30%. In a similar way, we produced the binary phenotype using a generalized linear model.

The phenotypes of 1,300 randomly selected Chinese (CHI) individuals were created in the target sample in the similar manner above. Here, the SNP effect sizes were set to $\text{β}\text{=}\text{b}\text{ω}\text{+}\text{δ}$, with $\text{δ}$ following a normal distribution with a mean of zero and a variance of $\sigma_{\delta}^{2}$. We specified $\omega$=0.1, 0.3, 0.5, 0.7, or 0.9, and $\sigma_{\delta}^{2}$=0.01 or 0.10. The summary statistics of the simulated auxiliary and target samples were obtained via a single-marker association analysis [[27](#_ENREF_27)]. We repeated 50 simulations for each scenario and evaluated the prediction accuracy by calculating *R*^2^ or area under curve (AUC).

# Quality control criteria for analyzed phenotypes and genotypes

We performed the following quality control criteria for analyzed phenotypes and diseases: (i) for a continuous phenotype, we excluded individuals with certain diseases (e.g., cancers) that likely affected this phenotype; (ii) we removed participants with the phenotypes more than five standard deviations from the average or those with missing values in phenotypes; (iii) the missing values of covariates (see blew) were filled by multivariate imputations by chained equations (MICE) under the assumption of missing at random; (iv) we conducted the standardization for continuous phenotypes to improve comparability across various datasets; (v) we carried out a rigorous pre-processing and quality control for genotypes and genetic variants under consideration [[15](#_ENREF_15), [28](#_ENREF_28), [29](#_ENREF_29)].

# Covariate selection for analyzed phenotypes

For the four lipid traits including high-density lipoprotein (HDL), low-density lipoprotein (LDL), triglycerides (TG), and total cholesterol (TC), we included sex, age, income, Townsend deprivation index (TDI), systolic blood pressure (SBP), diastolic blood pressure (DBP), body mass index (BMI), education, former smoking [[30](#_ENREF_30)], former drinking, physical activity [[31](#_ENREF_31)], and healthy diet score as covariates (Tables S1-S2).

For SBP and DBP, we first screened the participants who took antihypertensive medication and added 15 or 10 mm Hg, respectively [[32](#_ENREF_32)]. For them, we considered sex, income, TDI, age, BMI [[33](#_ENREF_33)], former smoking and drinking [[34](#_ENREF_34)], physical activity, and healthy diet score [[35](#_ENREF_35)] as covariates (Table S3).

For BMI, we included sex, age, income, education [[36](#_ENREF_36)], TDI, overall health rating, maternal smoking around birth, birth weight [[37](#_ENREF_37)], infant breastfeeding, comparative height size at age 10, comparative body size at age 10 [[38](#_ENREF_38)], former smoking [[30](#_ENREF_30)], physical activity [[31](#_ENREF_31)], and healthy diet score [[39](#_ENREF_39)] as covariates (Table S4).

For the three diseases, we only considered sex and age as covariates as only they were available from the Kaiser Permanente/UCSF Genetic Epidemiology of Adult Health and Ageing Study (GERA) [[40](#_ENREF_40), [41](#_ENREF_41)].

# Summary statistics of analyzed phenotypes

For the four lipid traits, we obtained their summary statistics from the most recent genome-wide association studies (GWAS) that were performed on African (AFR) (*n*=99.4K), East Asian (EAS) (*n*=146.5K) and EUR (*n*=1.32M) individuals from the Global Lipid Genetics Consortium ([Graham, et al., 2021](#_ENREF_1)). For the two blood pressures SBP and DBP), we obtained their summary statistics from the latest GWAS performed on individuals of AFR [[42](#_ENREF_42)], EAS [[43](#_ENREF_43)] and EUR [[44](#_ENREF_44)] ancestry, respectively. The summary statistics of BMI were available from GWASs implemented on individuals of EAS (*n*=158,284) [[45](#_ENREF_45)], AFR (*n*=13,976) [[42](#_ENREF_42)], or EUR (*n*=681,275) [[46](#_ENREF_46)] ancestry, respectively.

The summary statistics of asthma were obtained from GWASs implemented on individuals of EAS (*n*=31,577) [[47](#_ENREF_47)], AFR (*n*=3,015) [[48](#_ENREF_48)] and EUR (*n*=97,691) [[49](#_ENREF_49)] ancestry, respectively. The summary statistics of CAD were yielded from GWASs implemented on individuals of EAS (*n*=212,453) [[47](#_ENREF_47)] and EUR (*n*=1,165,690) [[50](#_ENREF_50)] ancestry, respectively. The summary statistics of T2D were available from GWASs implemented on individuals of EAS (*n*=254,373) [[51](#_ENREF_51)] and EUR (*n*=272,026) [[52](#_ENREF_52)] ancestry, respectively. See Table S5 for detailed information about these summary statistics datasets.

We performed rigorous quality control on these summary statistics [[15](#_ENREF_15), [28](#_ENREF_28), [29](#_ENREF_29)]: (i) removed all non-biallelic single nucleotide polymorphisms (SNPs); (ii) filtered out all SNPs with strand-ambiguous alleles; (iii) excluded SNPs without rs IDs, duplicated rs IDs or base pair position; (iv) removed SNPs not in the 1000 Genomes Project Phase 3 or SNPs whose base pair positions or allele did not match those in the 1000 Genomes Project Phase 3 [[3](#_ENREF_3)]; (v) excluded SNPs located within the major histocompatibility complex region (chr6: 28.5Mb-33.5Mb); (vi) removed all SNPs on chromosome X or Y; (vii) kept SNPs having MAF >1%.

# Trans-ethnic genetic similarity and heterogeneity across the EUR and non-EUR populations

## **Trans-ethnic genetic similarity across the EUR and non-EUR populations**

### *Trans-ethnic genetic overlap*

We first applied the recently proposed CONTO (Composite Null hypothesis test for Trans-ethnic genetic Overlap) method to explore the overall genetic overlap between traits from a perspective of composite null hypothesis testing [[53](#_ENREF_53)]. CONTO was originally developed for estimating gene-level trans-ethnic genetic overlap, we here utilized it at the SNP level by following the similar statistical principle.

To implement CONTO, for each phenotype in the EUR and non-EUR populations we classified relatively independent SNPs into four categories: (i) SNPs associated with neither of phenotypes with a probability of *P*_00_; (ii) SNPs only associated with the phenotype in the EUR population with a probability of *P*_10_; (iii) SNPs only associated with the phenotype in the non-EUR population with a probability of *P*_01_; (iv) SNPs associated with the phenotype in both populations with a probability of *P*_11_.

We estimated the probability of SNPs in each category and applied the likelihood ratio test to assess the significance of the trans-ethnic genetic overlap proportion, which was defined as GOP=*P*_11_/(*P*_10_+*P*_01_+*P*_11_). To alleviate the influence of LD on CONTO, we performed LD pruning (window=50 and *r*^2^=0.1) via PLINK based on the 1000 Genomes Project EUR-ancestry, EAS-ancestry or AFR-ancestry genotypes to generate uncorrected SNPs.

### *Trans-ethnic genetic correlation*

We applied popcorn [[54](#_ENREF_54)] to calculate the global trans-ethnic genetic correlation ($\rho$) of each phenotype between the EAS and EUR population or between the AFR and EUR population. Each genetic variant's trans-ethnic LD score was directly available at <https://github.com/brielin/popcorn>, which was estimated using genotypes from EAS (or AFR) and EUR individuals from the 1000 Genomes Project between the focal SNP and all surrounding ones within a 10Mb window [[3](#_ENREF_3)].

To produce an unbiased estimate, we did not restrict the boundary of the trans-ethnic genetic correlation. Thus, we likely obtained an estimated trans-ethnic genetic correlation that was larger than one or less than zero.

### *Linear regression for SNP effect sizes across populations*

We conducted a simple linear model without the intercept term for all selected SNPs of each phenotype by regressing effect sizes of SNPs in the non-EUR population on those in the EUR population [[55](#_ENREF_55)]. The regression slope provided an indicator for the relative magnitude of effect sizes for genetic influences between the two populations, and would be significantly different from zero if the EUR population were informative for the non-EUR population.

The *R*^2^ calculated in this linear model was applied to assess the prediction capability of SNP effect sizes of the phenotype in the EUR population on the SNP effect sizes of the phenotype in the non-EUR population.

## **Trans-ethnic genetic heterogeneity across the EUR and non-EUR populations**

### *Difference in LD and MAF of SNPs between the European and non-European populations*

To identify genetic heterogeneity in each phenotype, we here examined the difference in LD or MAF for selected SNPs between the two populations by performing a paired sample *t* test [[56](#_ENREF_56)]. We applied LD score to quantify LD and calculated the LD score for each SNP in the EAS, AFR and EUR populations with genotypes of individuals from EAS-ancestry, AFR-ancestry and EUR-ancestry in the 1000 Genomes Project, respectively.

### *Other differences in genetic background between the European and non-European populations*

It needed to highlight that other measures such as genetic effect direction, heritability and trans-ethnic genetic correlation also reflected the genetic heterogeneity simultaneously. For instance, a large proportion of SNP effect sizes in opposite directions, a large difference in heritability and a trans-ethnic genetic correlation estimate far from one indicated the evidence of genetic heterogeneity for phenotype across populations.

Particularly, for the SNP-based heritability (*h*^2^) we implemented an approximation normal test to examine the difference in heritability between the two populations [[55](#_ENREF_55)]

$u=(\hat{h}_{1}^{2}-\hat{h}_{2}^{2})/\sqrt[2]{{se}_{1}^{2}+{se}_{2}^{2}}$

where $\hat{h}_{1}^{2}$ and $\hat{h}_{2}^{2}$ are the heritability estimates for the EAS (or AFR) and EUR population, and *se*_1_ and *se*_2_ are the corresponding standard errors.

# Screen independent associated SNPs from summary statistics for summary-level transPGS

For summary-level transPGS, we screened SNPs in summary statistics of each population using the pruning (LD *r*^2^<0.1, 0.05, or 0.01, window size=100, 500, or 1,000kb) and thresholding (*P*<1×10^-4^, 1×10^-5^, 1×10^-6^, 1×10^-7^, or 1×10^-8^) method via PLINK [[57](#_ENREF_57)], with the genotypes of AFR, EAS or EUR individuals in the 1000 Genomes Project [[3](#_ENREF_3)] as the reference panel.

Afterwards, we obtained a total of 45 sets of SNPs across different conditions. Given a specific condition *j*, we calculated $\mathrm{PGS}_{j}=\sum_{p=1}^{P} G_{jp}\hat{\beta}_{jp}$ (*j*=1, …, 45), where $G_{jp}$ was the genotype (coded as 0, 1, or 2, indicating the number of effect allele) and $\hat{\beta}_{jp}$ was the effect size of the *p*^th^ SNP before or after transfer learning. We combined all PGSs via the generalized linear model $E\left( \boldsymbol{Y} \right)=g^{-1}(\mathbf{X}\boldsymbol{\alpha}+\sum_{j=1}^{45} \pi_{j}\mathrm{PGS}_{j})$, where *E* was the expectation, *g* was the link function, **X** denoted covariates such as age and sex with ***α*** the effect vector, and *π_j_* was the weight of PGS under distinct conditions. We utilized the ridge algorithm to estimate *π_j_* by assuming the every PGS had a predictive value on the phenotype.

We conducted the Monte Carlo cross-validation for phenotypic prediction [[16](#_ENREF_16)], with 80% of the participants as training data, while the remaining as the test data. We employed the training data to calculate the weight of PGS in real data applications. We examined the prediction performance in the test data. We performed 50 replications and calculated *R*^2^ or AUC to evaluate the predictive performance of the model for continuous or binary phenotypes, respectively.

# Simulation results for summary-level transPGS

We here reported several important points observed from the simulations. First, the performance of transPGS performed with additional auxiliary samples was much better than that obtained using only target samples (Figures S3-S4), with an average improvement of 10.4% in *R*^2^ for continuous phenotypes and 2.9% in AUC for binary phenotypes across all simulation scenarios.

Second, as could be expected, the prediction accuracy of transPGS was enhanced by the increase of the scale parameter *ω*. The correlation between *ω* and the improved prediction accuracy of summary-level transPGS was 0.910 for continuous phenotypes and 0.948 for binary phenotypes. For instance, when PVE=0.2 and $\sigma_{\delta}^{2}$=0.1, the increased prediction accuracy of continuous phenotypes varied from 0.9% for *ω*=0.1 to 27.1% for *ω*=0.9 after incorporating auxiliary samples, with an average increase of 11.8% across various values of *ω*; and the increased prediction accuracy of binary phenotypes varied from 0.3% for *ω*=0.1 to 8.6% for *ω*=0.9 after incorporating auxiliary samples, with an average increase of 5.4% across various values of *ω*. Particularly, when *ω*=0.9, transPGS for the target samples almost behaved as well as PGS for the auxiliary samples.

Third, with other parameters unchanged, the prediction performance improved as the PVE increased. For example, when PVE ranged from 0.1 to 0.3 and $\sigma_{\delta}^{2}$=0.1, the *R*^2^ of transPGS increased from 0.042 to 0.068 for continuous phenotypes. This observation further confirmed the previous conclusion that higher PVE led to more accurate prediction.

# Table S1. Participant characteristics of lipids in the AFR population of the UKB cohort ($\bar{x}\pm sd$or *n*).

| Variable | HDL | LDL | TC | TG |
| --- | --- | --- | --- | --- |
| Age | 53.82±8.45 | 52,50±8.08 | 52.49±8.08 | 52.50±8.08 |
| TDI | 0.25±3.13 | 2.65±3.43 | 2.65±3.43 | 2.66±3.43 |
| BMI | 27.25±4.41 | 29.52±5.43 | 29.52±5.39 | 29.51±5.39 |
| SBP | 139.28±21.12 | 142.58±21.61 | 142.58±21.62 | 142.0±21.62 |
| DBP | 85.23±11.41 | 87.59±12.41 | 87.61±12.41 | 87.62±12.40 |
| Income | 2.38±1.27 | 2.15±1.09 | 2.14±1.08 | 2.15±1.08 |
| healthy diet score | 3.32±1.09 | 3.32±1.28 | 3.24±1.07 | 3.21±1.28 |
| Sex (female/male) | 3,757/2,880 | 4,077/3,082 | 4,087/3,086 | 4,085/3,086 |
| Education (no/yes) | 4,092/2,545 | 4,381/2,778 | 4,410/2,763 | 4,436/2,735 |
| Former smoking (no/yes) | 3,821/2,816 | 4,121/3,038 | 4,130/3,043 | 4,129/3,042 |
| Former drinking (no/yes) | 6,239/398 | 6,729/430 | 6,741/432 | 6,739/432 |
| Physical activity (low/moderate/high) | 1,505/2,511/2,621 | 1,597/2,701/2,81 | 1,601/2,717/2,855 | 1,594/2,753/2,824 |

Table S2. Participant characteristics of lipids in the CHI population of the UKB cohort ($\bar{x}\pm sd$or *n*).

| Variable | HDL | LDL | TC | TG |
| --- | --- | --- | --- | --- |
| Age | 53.04±7.65 | 52,97±7.67 | 52.96±7.67 | 52.96±7.67 |
| TDI | -0.42±3.37 | -0.49±3.34 | -0.48±3.34 | -0.48±3.34 |
| BMI | 24.07±3.42 | 24.11±3.42 | 24.11±3.42 | 24.11±3.42 |
| SBP | 134.33±21.05 | 134.34±21.19 | 134.36±21.19 | 134.36±21.19 |
| DBP | 83.12±12.09 | 83.04±12.14 | 83.07±12.16 | 83.06±12.16 |
| Income | 2.63±1.13 | 2.63±1.12 | 2.63±1.12 | 2.63±1.12 |
| healthy diet score | 3.09±1.05 | 3.09±1.04 | 3.09±1.04 | 3.09±1.04 |
| Sex (female/male) | 812/490 | 888/536 | 889/537 | 888/537 |
| Education (no/yes) | 453/849 | 495/929 | 495/931 | 494/931 |
| Former smoking (no/yes) | 829/473 | 911/513 | 912/514 | 911/514 |
| Former drinking (no/yes) | 1,249/53 | 1,366/58 | 1,368/58 | 1,367/58 |
| Physical activity (low/moderate/high) | 231/682/389 | 250/742/433 | 251/742/433 | 251/741/433 |

# Table S3. Participant characteristics of adult blood pressure in the EUR, AFR and CHI populations of the UKB cohort ($\bar{x}\pm sd$or *n*).

| Variable | European | African | Chinese |
| --- | --- | --- | --- |
| SBP (mmHg) | 141.66±20.69 | 142.57±21.58 | 134.39±21.26 |
| DBP (mmHg) | 84.29±11.26 | 87.60±12.38 | 83.07±12.15 |
| Age (year) | 57.31±8.03 | 52.46±8.06 | 52.98±7.67 |
| TDI | -1.46±2.99 | 2.65±3.44 | -0.42±3.34 |
| Income | 2.60±1.20 | 2.14±1.09 | 2.62±1.12 |
| healthy diet score | 3.14±1.26 | 3.22±1.28 | 3.09±1.04 |
| BMI (kg/m^2^) | 27.39±4.76 | 29.50±5.36 | 24.11±3.44 |
| Overall health rating | 2.12±0.73 | 2.33±0.76 | 2.21±0.67 |
| Sex (female/male) | 248,925/209,801 | 4,340/3,274 | 938/562 |
| Education (no/yes) | 286,155/172,571 | 4,703/2,911 | 524/976 |
| Former smoking (no/yes) | 178,519/280,207 | 4,400/3,214 | 969/531 |
| Former drinking (no/yes) | 441,397/17,329 | 7,168/446 | 1,440/60 |
| physical activity (low/moderate/high) | 86,628/187,603/184,495 | 1,704/2,919/2,991 | 269/778/453 |

# Table S4. Participant characteristics of adult BMI in the EUR, AFR and CHI populations of the UKB cohort ($\bar{x}\pm sd$or *n*).

| Variable | European | African | Chinese |
| --- | --- | --- | --- |
| BMI (kg/m^2^) | 26.65±4.34 | 29.49±5.38 | 24.11±3.44 |
| Age (year) | 57.23±8.17 | 53.43±8.06 | 54.26±7.57 |
| Income | 2.74±1.11 | 2.13±1.07 | 2.60±1.11 |
| TDI | -1.54±2.94 | 2.66±3.44 | -0.49±3.35 |
| Overall health rating | 2.01±0.70 | 2.33±0.76 | 2.21±0.67 |
| Birth weight (kg) | 3.34±0.50 | 3.27±0.70 | 3.13±0.38 |
| healthy diet score | 3.11±1.25 | 3.15±1.29 | 2.99±1.21 |
| Sex (female/male) | 153,237/127,338 | 4,297/3,236 | 934/561 |
| Education (no/yes) | 181,202/99,253 | 4,047/3,486 | 524/971 |
| Maternal smoking around birth (no/yes) | 207,829/72,746 | 7,257/276 | 1,404/91 |
| Infant breastfeeding (no/yes) | 65,563/215,012 | 336/7,197 | 296/1,199 |
| Comparative height size at age 10 (average/shorter/taller) | 153,707/56,233/70,635 | 4,345/1,406/1,782 | 342/217/936 |
| Comparative body size at age 10 (average/thinner/plumper) | 148,385/90,555/41,635 | 3,706/2,631/1,196 | 534/148/813 |
| Ever smoked (no/yes) | 112,869/167,706 | 4,304/3,229 | 969/526 |
| physical activity (low/moderate/high) | 67,671/133,564/79,340 | 2,399/3,387/1,747 | 461/689/345 |

# Table S5. Information of GWAS summary statistics performed on AFR-ancestry, EUR-ancestry or EAS-ancestry individuals.

| phenotype | *k*_1_ | *k*_2_ | *N* | Reference |
| --- | --- | --- | --- | --- |
| **AFR** |  |  |  |  |
| HDL | 31,795,570 | 12,300,802 | 99.4K | [[58](#_ENREF_58)] |
| LDL | 31,627,319 | 12,299,150 | 99.4K | [[58](#_ENREF_58)] |
| TC | 31,916,286 | 12,302,442 | 99.4K | [[58](#_ENREF_58)] |
| TG | 31,759,448 | 12,301,044 | 99.4K | [[58](#_ENREF_58)] |
| SBP | 14,261,064 | 11,982,649 | 13,613 | [[42](#_ENREF_42)] |
| DBP | 14,290,419 | 12,006,436 | 13,618 | [[42](#_ENREF_42)] |
| BMI | 14,265,477 | 11,985,310 | 13,976 | [[42](#_ENREF_42)] |
| CAD | / | / | / | / |
| T2D | / | / | / | / |
| asthma | 336,563 | 335,261 | 3,015 | [[42](#_ENREF_42)] |
| **EAS** |  |  |  |  |
| HDL | 16,536,398 | 6,225,849 | 146.5K | [[58](#_ENREF_58)] |
| LDL | 15,226,956 | 6,225,823 | 146.5K | [[58](#_ENREF_58)] |
| TC | 16,936,802 | 6,225,868 | 146.5K | [[58](#_ENREF_58)] |
| TG | 15,255,562 | 6,225,837 | 146.5K | [[58](#_ENREF_58)] |
| SBP | 5,961,104 | 4,816,166 | 136,597 | [[43](#_ENREF_43)] |
| DBP | 5,961,104 | 4,816,166 | 136,615 | [[43](#_ENREF_43)] |
| BMI | 5,961,104 | 4,816,166 | 158,284 | [[45](#_ENREF_45)] |
| CAD | 8,678,731 | 5,369,907 | 212,453 | [[47](#_ENREF_47)] |
| T2D | 7,867,899 | 6,043,761 | 254,373 | [[51](#_ENREF_51)] |
| asthma | 7,867,872 | 5,336,505 | 31,577 | [[47](#_ENREF_47)] |
| **EUR** |  |  |  |  |
| HDL | 46,150,908 | 6,656,751 | 1.32M | [[58](#_ENREF_58)] |
| LDL | 47,006,483 | 6,656,756 | 1.32M | [[58](#_ENREF_58)] |
| TC | 47,196,261 | 6,656,723 | 1.32M | [[58](#_ENREF_58)] |
| TG | 46,513,217 | 6,656,754 | 1.32M | [[58](#_ENREF_58)] |
| SBP | 7,080,765 | 5,821,910 | 757,601 | [[44](#_ENREF_44)] |
| DBP | 7,009,209 | 5,872,908 | 757,601 | [[44](#_ENREF_44)] |
| BMI | 2,336,269 | 1,955,635 | 681,275 | [[46](#_ENREF_46)] |
| CAD | 20,073,070 | 7,072,998 | 1,165,690 | [[50](#_ENREF_50)] |
| T2D | 18,409,439 | 6,471,860 | 272,026 | [[52](#_ENREF_52)] |
| asthma | 8,985,294 | 6,787,036 | 97,691 | [[49](#_ENREF_49)] |

Note: *k*_1_: represents the number of SNPs before quality control; *k*_2_: represents the number of SNPs after quality control; *N*: represents the number of samples. “/”: no summary statistics data are available.

# Table S6. Trans-ethnic genetic overlap for the ten phenotypes between the EAS/AFR and EUR populations (LD pruning based on EAS-ancestry or AFR-ancestry genotypes).

| phenotype | *P*_00_ (*se*) | *P*_10_ (*se*) | *P*_01_ (*se*) | *P*_11_ (*se*) | GOP | LRT (*P*) |
| --- | --- | --- | --- | --- | --- | --- |
| **EAS** |  |  |  |  |  |  |
| HDL | 0.784 (0.003) | 0.000 (0.002) | 0.180 (0.003) | 0.036 (0.002) | 0.168 | 708.5 (4.31×10^-156^) |
| LDL | 0.922 (0.001) | 0.000 (0.001) | 0.064 (0.001) | 0.014 (0.001) | 0.181 | 1625.2 (1.80×10^-366^) |
| TC | 0.909 (0.001) | 0.000 (0.001) | 0.070 (0.001) | 0.021 (0.001) | 0.232 | 2953.3 (1.80×10^-366^) |
| TG | 0.836 (0.002) | 0.000 (0.000) | 0.159 (0.001) | 0.006 (0.000) | 0.034 | 845.5 (7.09×10^-186^) |
| SBP | 0.709 (0.003) | 0.014 (0.003) | 0.195 (0.004) | 0.083 (0.004) | 0.284 | 717.5 (4.65×10^-158^) |
| DBP | 0.726 (0.004) | 0.014 (0.003) | 0.168 (0.005) | 0.092 (0.005) | 0.337 | 678.7 (1.26×10^-149^) |
| BMI | 0.564 (0.006) | 0.032 (0.005) | 0.194 (0.007) | 0.210 (0.007) | 0.483 | 739.7 (7.05×10^-163^) |
| CAD | 0.878 (0.002) | 0.000 (0.001) | 0.076 (0.002) | 0.046 (0.002) | 0.376 | 1708.7 (1.80×10^-366^) |
| T2D | 0.800 (0.003) | 0.000 (0.002) | 0.120 (0.003) | 0.080 (0.002) | 0.402 | 2898.9 (1.80×10^-366^) |
| asthma | 0.884 (0.004) | 0.000 (0.003) | 0.074 (0.005) | 0.042 (0.004) | 0.360 | 317.1 (6.35×10^-71^) |
| **AFR** |  |  |  |  |  |  |
| HDL | 0.788 (0.001) | 0.005 (0.001) | 0.175 (0.001) | 0.032 (0.001) | 0.153 | 1980.5 (1.80×10^-366^) |
| LDL | 0.898 (0.001) | 0.007 (0.001) | 0.074 (0.001) | 0.021 (0.001) | 0.210 | 2798.0 (1.80×10^-366^) |
| TC | 0.876 (0.001) | 0.015 (0.001) | 0.083 (0.001) | 0.026 (0.001) | 0.213 | 2891.5 (1.80×10^-366^) |
| TG | 0.790 (0.002) | 0.024 (0.001) | 0.141 (0.001) | 0.046 (0.001) | 0.216 | 1421.1 (1.12×10^-308^) |
| SBP | 0.771 (0.041) | 0.168 (0.077) | 0.018 (0.041) | 0.043 (0.077) | 0.189 | 3.2 (7.15×10^-2^) |
| DBP | 0.727 (0.096) | 0.025 (0.096) | 0.238 (0.041) | 0.010 (0.041) | 0.038 | 0.3 (5.77×10^-1^) |
| BMI | 0.551 (0.026) | 0.036 (0.026) | 0.362 (0.033) | 0.051 (0.033) | 0.114 | 2.7 (9.75×10^-2^) |
| asthma | 0.896 (8.496) | 0.009 (8.496) | 0.095 (4.489) | 0.001 (4.490) | 0.007 | 0.1 (9.26×10^-1^) |

Note: the trans-ethnic genetic overlap was estimated by CONTO; *P*_11_ denotes the proportion of SNPs associated with the phenotype in both populations; *P*_10_ denotes the probability of SNPs only associated with the phenotype in the EUR population; *P*_01_ denotes the probability of SNPs only associated with the phenotype in the non-EUR population; GOP=*P*_11_/(*P*_10_+*P*_01_+*P*_11_) represents the trans-ethnic genetic overlap proportion of SNPs related to the phenotype in both populations against the proportion of SNPs related to the phenotype in at least one population; the LRT statistic and *P* for the significance of GOP are also given.

# Table S7. Trans-ethnic genetic overlap for the ten phenotypes between the EAS/AFR and EUR populations (LD pruning based on EUR-ancestry genotypes).

| phenotype | *P*_00_ (*se*) | *P*_10_ (*se*) | *P*_01_ (*se*) | *P*_11_ (*se*) | GOP | LRT (*P*) |
| --- | --- | --- | --- | --- | --- | --- |
| **EAS** |  |  |  |  |  |  |
| HDL | 0.830 (0.001) | 0.142 (0.001) | 0.000 (0.001) | 0.027 (0.001) | 0.161 | 2409.5 (1.80×10^-366^) |
| LDL | 0.930 (0.001) | 0.054 (0.001) | 0.000 (0.000) | 0.016 (0.001) | 0.234 | 2442.0 (1.80×10^-366^) |
| TC | 0.916 (0.001) | 0.057 (0.001) | 0.000 (0.000) | 0.027 (0.001) | 0.317 | 4430.9 (1.80×10^-366^) |
| TG | 0.851 (0.001) | 0.132 (0.001) | 0.000 (0.001) | 0.016 (0.001) | 0.111 | 1534.5 (1.80×10^-366^) |
| SBP | 0.728 (0.004) | 0.138 (0.004) | 0.023 (0.003) | 0.112 (0.004) | 0.410 | 1144.9 (5.70×10^-251^) |
| DBP | 0.743 (0.004) | 0.137 (0.005) | 0.020 (0.003) | 0.100 (0.005) | 0.391 | 928.4 (6.54×10^-204^) |
| BMI | 0.598 (0.005) | 0.148 (0.007) | 0.040 (0.005) | 0.215 (0.007) | 0.534 | 907.5 (2.32×10^-199^) |
| CAD | 0.884 (0.002) | 0.047 (0.002) | 0.008 (0.002) | 0.060 (0.002) | 0.521 | 2302.5 (1.80×10^-366^) |
| T2D | 0.812 (0.002) | 0.092 (0.002) | 0.003 (0.002) | 0.093 (0.002) | 0.493 | 3427.4 (1.80×10^-366^) |
| asthma | 0.887 (0.005) | 0.045 (0.006) | 0.006 (0.004) | 0.062 (0.005) | 0.543 | 334.9 (8.07×10^-75^) |
| **AFR** |  |  |  |  |  |  |
| HDL | 0.824 (0.002) | 0.132 (0.002) | 0.011 (0.001) | 0.034 (0.001) | 0.192 | 1090.3 (4.15×10^-239^) |
| LDL | 0.921 (0.001) | 0.053 (0.001) | 0.008 (0.001) | 0.018 (0.001) | 0.233 | 1676.9 (1.80×10^-366^) |
| TC | 0.900 (0.001) | 0.060 (0.001) | 0.017 (0.001) | 0.024 (0.001) | 0.238 | 1724.0 (1.80×10^-366^) |
| TG | 0.820 (0.002) | 0.096 (0.002) | 0.035 (0.002) | 0.049 (0.002) | 0.274 | 921.3 (2.26×10^-202^) |
| SBP | 0.747 (0.010) | 0.206 (0.014) | 0.016 (0.010) | 0.031 (0.014) | 0.124 | 17.1 (3.63×10^-5^) |
| DBP | 0.772 (0.013) | 0.200 (0.023) | 0.008 (0.013) | 0.020 (0.022) | 0.088 | 7.8 (5.20×10^-3^) |
| BMI | 0.636 (0.011) | 0.310 (0.019) | 0.010 (0.010) | 0.043 (0.019) | 0.119 | 11.8 (6.02×10^-4^) |
| asthma | 0.879 (12.643) | 0.112 (7.339) | 0.009 (12.643) | 0.001 (7.340) | 0.007 | 0.1 (9.38×10^-1^) |

Note: the trans-ethnic genetic overlap was estimated by CONTO; *P*_11_ denotes the proportion of SNPs associated with the phenotype in both populations; *P*_10_ denotes the probability of SNPs only associated with the phenotype in the EUR population; *P*_01_ denotes the probability of SNPs only associated with the phenotype in the non-EUR population; GOP=*P*_11_/(*P*_10_+*P*_01_+*P*_11_) represents the trans-ethnic genetic overlap proportion of SNPs related to the phenotype in both populations against the proportion of SNPs related to the phenotype in at least one population; the LRT statistic and *P* for the significance of GOP are also given.

# Table S8. Estimates of heritability and trans-ethnic genetic correlation for the ten phenotypes between the non-EUR and EUR populations.

| phenotype | $\hat{h}_{1}^{2}$ (*se*_1_) | $\hat{h}_{2}^{2}$ (*se*_2_) | $P_{\Delta\hat{h}^{2}}$ | $\hat{\rho}$ (*se*) | $P_{\hat{\rho}}$ | slope | *R*^2^ |
| --- | --- | --- | --- | --- | --- | --- | --- |
| **EAS** |  |  |  |  |  |  |  |
| HDL | 0.061 (0.014) | 0.050 (0.016) | 5.88×10^-1^ | 1.138 (0.146) | 3.47×10^-1^ | 0.324 | 0.477 |
| LDL | 0.031 (0.017) | 0.040 (0.018) | 7.43×10^-1^ | 0.898 (0.127) | 4.19×10^-1^ | 0.303 | 0.593 |
| TC | 0.049 (0.013) | 0.048 (0.010) | 9.46×10^-1^ | 0.959 (0.069) | 5.48×10^-1^ | 0.305 | 0.470 |
| TG | 0.073 (0.015) | 0.082 (0.023) | 7.30×10^-1^ | 1.044 (0.055) | 4.19×10^-1^ | 0.343 | 0.520 |
| SBP | 0.087 (0.008) | 0.050 (0.005) | 4.44×10^-5^ | 0.897 (0.054) | 5.84×10^-2^ | 0.185 | 0.241 |
| DBP | 0.086 (0.007) | 0.039 (0.004) | 1.97×10^-8^ | 0.974 (0.080) | 7.47×10^-1^ | 0.153 | 0.293 |
| BMI | 0.154 (0.010) | 0.115 (0.008) | 2.23×10^-3^ | 0.843 (0.036) | 1.12×10^-5^ | 0.223 | 0.372 |
| CAD | 0.019 (0.002) | 0.042 (0.005) | 7.93×10^-6^ | 0.861 (0.076) | 4.69×10^-1^ | -0.165 | 0.383 |
| T2D | 0.029 (0.002) | 0.048 (0.005) | 1.21×10^-3^ | 0.927 (0.039) | 5.70×10^-2^ | 0.104 | 0.419 |
| asthma | 0.024 (0.003) | 0.012 (0.002) | 1.02×10^-3^ | 0.743 (1.918) | 5.50×10^-2^ | 0.052 | 0.332 |
| **AFR** |  |  |  |  |  |  |  |
| HDL | 0.029 (0.012) | 0.047 (0.040) | 6.63×10^-1^ | 1.291 (0.448) | 5.16×10^-1^ | 0.174 | 0.500 |
| LDL | 0.016 (0.014) | 0.021 (0.015) | 7.90×10^-1^ | 0.670 (0.221) | 1.36×10^-1^ | 0.185 | 0.387 |
| TC | 0.048 (0.013) | 0.025 (0.009) | 1.28×10^-1^ | 0.674 (0.144) | 9.54×10^-1^ | 0.176 | 0.344 |
| TG | 0.072 (0.015) | 0.029 (0.007) | 8.98×10^-3^ | 0.835 (0.102) | 5.77×10^-1^ | 0.178 | 0.499 |
| SBP | 0.088 (0.008) | 0.012 (0.019) | 2.97×10^-4^ | 0.862 (0.756) | 8.55×10^-1^ | 0.112 | 0.014 |
| DBP | 0.011 (0.021) | 0.079 (0.007) | 1.72×10^-3^ | 1.206 (1.179) | 8.62×10^-1^ | 0.106 | 0.023 |
| BMI | 0.153 (0.010) | 0.015 (0.020) | 7.86×10^-10^ | 0.412 (0.318) | 4.94×10^-4^ | 0.059 | 0.065 |
| asthma | 0.026 (0.003) | 0.091 (0.047) | 1.66×10^-1^ | 0.411 (0.194) | 2.44×10^-3^ | 0.045 | 0.209 |

Note: $P_{\Delta\hat{h}^{2}}$ denotes the *P* value available via an approximate normal test for examining the difference between $\hat{h}_{1}^{2}$ and $\hat{h}_{2}^{2}$; $P_{\hat{\rho}}$ denotes the *P* value for the significance of whether $\hat{\rho}$ is different from one. For both $P_{\Delta\hat{h}^{2}}$ and $P_{\hat{\rho}}$, the significant difference after multiple-comparison correction is shown in bold.

# Table S9. MAF and LD comparison for selected SNPs of the ten phenotypes between the EAS and EUR populations or the AFR and EUR populations.

| phenotype | Difference of MAF | MAF [*t* (*P*)] | Difference of LD | LD [*t* (*P*)] |
| --- | --- | --- | --- | --- |
| **EAS** |  |  |  |  |
| HDL | -0.02 | -5.00 (7.69×10^-7^) | -6.05 | -2.80 (5.26×10^-3^) |
| LDL | -0.02 | -2.59 (9.90×10^-3^) | -6.04 | -3.03 (2.58×10^-3^) |
| TC | -0.02 | -4.69 (3.48×10^-6^) | -6.26 | -2.58 (1.03×10^-2^) |
| TG | -0.02 | -3.45 (6.18×10^-4^) | -6.27 | -4.09 (5.23×10^-5^) |
| SBP | -0.02 | -3.74 (2.13×10^-4^) | -5.77 | -3.71 (2.41×10^-4^) |
| DBP | -0.02 | -4.39 (1.53×10^-5^) | -6.65 | -1.83 (6.77×10^-2^) |
| BMI | -0.02 | 9.05 (3.88×10^-18^) | -7.09 | -4.62 (4.87×10^-6^) |
| CAD | -0.02 | -1.51 (1.34×10^-1^) | -6.21 | -2.79 (5.78×10^-3^) |
| T2D | -0.01 | 1.14 (2.54×10^-1^) | -5.88 | -1.93 (5.50×10^-2^) |
| asthma | -0.01 | -3.00 (5.14×10^-3^) | -7.56 | -1.76 (8.82×10^-2^) |
| **AFR** |  |  |  |  |
| HDL | -0.02 | -5.37 (1.24×10^-7^) | -52.35 | -11.83 (1.22×10^-28^) |
| LDL | -0.02 | -2.76 (6.12×10^-3^) | -52.04 | -18.35 (3.00×10^-55^) |
| TC | -0.02 | -5.09 (5.01×10^-7^) | -52.35 | -16.98 (1.44×10^-51^) |
| TG | -0.02 | -4.87 (1.61×10^-6^) | -52.74 | -17.95 (1.07×10^-52^) |
| SBP | -0.02 | 9.84 (2.98×10^-20^) | -56.28 | -8.83 (5.78×10^-17^) |
| DBP | -0.02 | 9.31 (1.37×10^-18^) | -53.96 | -9.04 (1.11×10^-17^) |
| BMI | -0.03 | 5.06 (6.93×10^-88^) | -44.70 | -26.61 (5.33×10^-95^) |
| asthma | -0.03 | -4.10 (2.66×10^-3^) | -42.57 | -4.82 (9.47×10^-4^) |

Note: Differences in LD and MAF of selected SNPs between the EUR and non-EUR populations were compared by a paired-sample *t* test, with the *t* statistic and *P* value shown.

# Table 10. Estimated *R*^2^ or AUC of prediction models for the seven continuous phenotypes and three diseases in the UKB CHI population and the GERA EAS population before and after transfer learning using individual-level data with SNPs screened via a threshold of 5×10^-6^.

| phenotype | original model | | |  | trans-ethnic model | |  | individual-level transPGS | |
| --- | --- | --- | --- | --- | --- | --- | --- | --- | --- |
|  | Z (*se*) | Z+G (*se*) | gain (%) |  | Z+G (*se*) | gain (%) |  | Z+G (*se*) | gain (%) |
| UKB (CHI) |  |  |  |  |  |  |  |  |  |
| BMI | 0.003 (0.003) | 0.004 (0.008) | 3.81 |  | 0.001 (0.003) | -61.88 |  | 0.005 (0.001) | 33.57 |
| DBP | 0.092 (0.003) | 0.097 (0.005) | 5.34 |  | 0.089 (0.004) | -2.78 |  | 0.169 (0.002) | 84.20 |
| HDL | 0.013 (0.001) | 0.013 (0.008) | 2.14 |  | 0.007 (0.002) | -44.74 |  | 0.019 (0.007) | 47.51 |
| LDL | 0.011 (0.008) | 0.013 (0.003) | 17.07 |  | 0.012 (0.008) | 6.02 |  | 0.014 (0.002) | 24.95 |
| SBP | 0.187 (0.002) | 0.188 (0.003) | 0.20 |  | 0.185 (0.005) | -1.01 |  | 0.269 (0.004) | 43.54 |
| TC | 0.016 (0.007) | 0.017 (0.007) | 7.88 |  | 0.016 (0.008) | 3.85 |  | 0.018 (0.003) | 8.35 |
| TG | 0.037 (0.004) | 0.037 (0.008) | 0.16 |  | 0.037 (0.017) | 0.25 |  | 0.053 (0.008) | 41.68 |
| GERA (EAS) |  |  |  |  |  |  |  |  |  |
| CAD | 0.793 (0.003) | 0.795 (0.004) | 0.30 |  | 0.605 (0.004) | -23.63 |  | 0.795 (0.012) | 0.35 |
| T2D | 0.703 (0.004) | 0.722 (0.008) | 2.73 |  | 0.533 (0.007) | -24.20 |  | 0.723 (0.005) | 2.82 |
| Asthma | 0.547 (0.006) | 0.585 (0.007) | 6.85 |  | 0.516 (0.001) | -5.72 |  | 0.585 (0.002) | 6.92 |

Note: Z: covariates only prediction model; original model: prediction model with covariate and PGS; trans-ethnic model: prediction model with covariate and PGS calculated with SNP effect sizes of the EUR population; transPGS: prediction model for the CHI or EAS population after transfer learning of SNP effect sizes by utilizing the genetic information of the EUR population; gain of prediction accuracy was calculated by comparing the PGS model with the covariates only prediction model.

# Table S11. Estimated *R*^2^ or AUC of prediction models for the seven continuous phenotypes and three diseases in the UKB AFR population and the GERA AFR population before and after transfer learning using individual-level data with SNPs screened via a threshold of 5×10^-6^.

| phenotype | original model | | |  | trans-ethnic model | |  | individual-level transPGS | |
| --- | --- | --- | --- | --- | --- | --- | --- | --- | --- |
|  | Z (*se*) | Z+G (*se*) | gain (%) |  | Z+G (*se*) | gain (%) |  | Z+G (*se*) | gain (%) |
| UKB (AFR) |  |  |  |  |  |  |  |  |  |
| BMI | 0.075 (0.012) | 0.077 (0.012) | 2.71 |  | 0.075 (0.013) | 0.02 |  | 0.097 (0.016) | 28.59 |
| DBP | 0.083 (0.013) | 0.084 (0.018) | 1.11 |  | 0.083 (0.027) | -0.48 |  | 0.103 (0.018) | 23.70 |
| HDL | 0.188 (0.008) | 0.195 (0.004) | 3.43 |  | 0.194 (0.002) | 2.87 |  | 0.197 (0.004) | 4.45 |
| LDL | 0.057 (0.012) | 0.060 (0.024) | 5.25 |  | 0.052 (0.013) | -9.28 |  | 0.062 (0.019) | 8.34 |
| SBP | 0.163 (0.011) | 0.166 (0.009) | 1.87 |  | 0.163 (0.011) | 0.00 |  | 0.180 (0.011) | 10.41 |
| TC | 0.042 (0.015) | 0.043 (0.010) | 3.19 |  | 0.036 (0.016) | -12.83 |  | 0.046 (0.021) | 9.80 |
| TG | 0.074 (0.008) | 0.075 (0.008) | 1.73 |  | 0.074 (0.025) | 0.94 |  | 0.075 (0.028) | 2.21 |
| CAD | 0.680 (0.005) | 0.690 (0.019) | 1.47 |  | 0.649 (0.003) | -4.52 |  | 0.691 (0.003) | 1.56 |
| T2D | 0.669 (0.007) | 0.670 (0.003) | 0.04 |  | 0.601 (0.007) | -10.26 |  | 0.693 (0.008) | 3.54 |
| Asthma | 0.566 (0.010) | 0.575 (0.015) | 1.50 |  | 0.563 (0.010) | -0.61 |  | 0.575 (0.012) | 1.60 |
| GERA (AFR) |  |  |  |  |  |  |  |  |  |
| Asthma | 0.582 (0.011) | 0.593 (0.010) | 1.85 |  | 0.517 (0.015) | -11.27 |  | 0.594 (0.014) | 2.02 |
| CAD | 0.781 (0.006) | 0.781 (0.007) | 0.04 |  | 0.622 (0.003) | -20.29 |  | 0.793 (0.006) | 1.59 |
| T2D | 0.601 (0.012) | 0.651 (0.008) | 8.27 |  | 0.528 (0.007) | -12.12 |  | 0.651 (0.017) | 8.35 |

Note: Z: covariates only prediction model; original model: prediction model with covariate and PGS; trans-ethnic model: prediction model with covariate and PGS calculated with SNP effect sizes of the EUR population; transPGS: prediction model for the AFR population after transfer learning of SNP effect sizes by utilizing the genetic information of the EUR population; gain of prediction accuracy was calculated by comparing the PGS model with the covariates only prediction model.

# Table S12. Estimated R2 or AUC of prediction models for the seven continuous phenotypes and three diseases in the UKB CHI population and the GERA EAS population before and after transfer learning using individual-level data with SNPs screened via a threshold of 5×10^-7^.

| phenotype | original model | | |  | trans-ethnic model | |  | individual-level transPGS | |
| --- | --- | --- | --- | --- | --- | --- | --- | --- | --- |
|  | Z (*se*) | Z+G (*se*) | gain (%) |  | Z+G (*se*) | gain (%) |  | Z+G (*se*) | gain (%) |
| UKB (CHI) |  |  |  |  |  |  |  |  |  |
| BMI | 0.025 (0.003) | 0.025 (0.017) | 0.02 |  | 0.024 (0.003) | -0.63 |  | 0.027 (0.004) | 7.18 |
| DBP | 0.109 (0.004) | 0.116 (0.018) | 6.35 |  | 0.106 (0.003) | -2.91 |  | 0.168 (0.003) | 54.16 |
| HDL | 0.209 (0.008) | 0.209 (0.002) | 0.23 |  | 0.209 (0.012) | 0.16 |  | 0.292 (0.006) | 39.66 |
| LDL | 0.009 (0.009) | 0.010 (0.023) | 12.38 |  | 0.009 (0.008) | -5.10 |  | 0.012 (0.009) | 22.71 |
| SBP | 0.210 (0.014) | 0.214 (0.012) | 1.91 |  | 0.206 (0.002) | -1.61 |  | 0.270 (0.005) | 28.78 |
| TC | 0.020 (0.008) | 0.022 (0.006) | 11.30 |  | 0.021 (0.007) | 5.61 |  | 0.023 (0.009) | 14.26 |
| TG | 0.050 (0.021) | 0.051 (0.008) | 0.41 |  | 0.051 (0.014) | 1.17 |  | 0.082 (0.015) | 62.45 |
| GERA (EAS) |  |  |  |  |  |  |  |  |  |
| CAD | 0.793 (0.007) | 0.795 (0.001) | 0.28 |  | 0.627 (0.003) | -20.91 |  | 0.795 (0.008) | 0.30 |
| T2D | 0.704 (0.008) | 0.721 (0.009) | 2.43 |  | 0.663 (0.007) | -19.95 |  | 0.721 (0.015) | 2.46 |
| Asthma | 0.548 (0.002) | 0.584 (0.016) | 6.50 |  | 0.470 (0.009) | -14.18 |  | 0.584 (0.007) | 6.60 |

Note: Z: covariates only prediction model; original model: prediction model with covariate and PGS; trans-ethnic model: prediction model with covariate and PGS calculated with SNP effect sizes of the EUR population; transPGS: prediction model for the CHI or EAS population after transfer learning of SNP effect sizes by utilizing the genetic information of the EUR population; gain of prediction accuracy was calculated by comparing the PGS model with the covariates only prediction model.

# Table S13. Estimated R2 or AUC of prediction models for the seven continuous phenotypes and three diseases in the UKB AFR population and the GERA AFR population before and after transfer learning using individual-level data with SNPs screened via a threshold of 5×10^-7^.

| phenotype | original model | | |  | trans-ethnic model | |  | individual-level transPGS | |
| --- | --- | --- | --- | --- | --- | --- | --- | --- | --- |
|  | Z (*se*) | Z+G (*se*) | gain (%) |  | Z+G (*se*) | gain (%) |  | Z+G (*se*) | gain (%) |
| UKB (AFR) |  |  |  |  |  |  |  |  |  |
| BMI | 0.083 (0.007) | 0.083 (0.002) | 1.25 |  | 0.084 (0.012) | 1.28 |  | 0.097 (0.011) | 16.70 |
| DBP | 0.085 (0.019) | 0.085 (0.007) | 0.97 |  | 0.084 (0.004) | -0.32 |  | 0.103 (0.003) | 20.49 |
| HDL | 0.188 (0.003) | 0.188 (0.006) | 6.80 |  | 0.199 (0.023) | 5.60 |  | 0.202 (0.012) | 6.96 |
| LDL | 0.007 (0.014) | 0.057 (0.002) | 7.69 |  | 0.052 (0.009) | -9.60 |  | 0.063 (0.003) | 10.95 |
| SBP | 0.167 (0.011) | 0.167 (0.008) | 0.75 |  | 0.166 (0.010) | -0.38 |  | 0.180 (0.027) | 7.64 |
| TC | 0.016 (0.007) | 0.048 (0.002) | 4.51 |  | 0.048 (0.011) | 4.36 |  | 0.048 (0.013) | 4.77 |
| TG | 0.074 (0.008) | 0.078 (0.009) | 6.72 |  | 0.075 (0.003) | 5.77 |  | 0.080 (0.004) | 7.44 |
| CAD | 0.689 (0.019) | 0.690 (0.010) | 0.05 |  | 0.647 (0.027) | -6.19 |  | 0.691 (0.003) | 0.05 |
| T2D | 0.650 (0.004) | 0.660 (0.019) | 1.42 |  | 0.601 (0.013) | -8.93 |  | 0.671 (0.005) | 1.49 |
| Asthma | 0.566 (0.014) | 0.567 (0.000) | 0.86 |  | 0.566 (0.004) | 0.14 |  | 0.572 (0.002) | 1.12 |
| GERA (AFR) |  |  |  |  |  |  |  |  |  |
| Asthma | 0.483 (0.007) | 0.483 (0.002) | 1.25 |  | 0.439 (0.017) | -7.70 |  | 0.500 (0.007) | 3.10 |
| CAD | 0.685 (0.019) | 0.685 (0.007) | 0.97 |  | 0.621 (0.013) | -20.54 |  | 0.783 (0.002) | 14.31 |
| T2D | 0.588 (0.003) | 0.588 (0.006) | 0.80 |  | 0.525 (0.004) | -12.70 |  | 0.654 (0.002) | 8.78 |

Note: Z: covariates only prediction model; original model: prediction model with covariate and PGS; trans-ethnic model: prediction model with covariate and PGS calculated with SNP effect sizes of the EUR population; transPGS: prediction model for the AFR population after transfer learning of SNP effect sizes by utilizing the genetic information of the EUR population; gain of prediction accuracy was calculated by comparing the PGS model with the covariates only prediction model.

# Table S14. Estimated *R*^2^ or AUC of prediction models for the seven continuous phenotypes and three diseases in the UKB CHI population and the GERA EAS population before and after transfer learning with individual-level data.

| phenotype | EUR | |  | AFR | |
| --- | --- | --- | --- | --- | --- |
|  | transPGS (*se*) | gain (%) |  | transPGS (*se*) | gain (%) |
| UKB (CHI) |  |  |  |  |  |
| HDL | 0.245 (0.021) | 32.43 |  | 0.200 (0.029) | -18.37 |
| LDL | 0.066 (0.001) | 175.00 |  | 0.035 (0.001) | -46.97 |
| TC | 0.047 (0.003) | 161.11 |  | 0.027 (0.004) | -42.55 |
| TG | 0.056 (0.017) | 55.56 |  | 0.042 (0.015) | -25.00 |
| SBP | 0.251 (0.022) | 22.44 |  | 0.224 (0.027) | -10.76 |
| DBP | 0.121 (0.018) | 3.42 |  | 0.121 (0.021) | 0.00 |
| BMI | 0.062 (0.011) | 16.98 |  | 0.059 (0.012) | -4.84 |
| GERA (EAS) |  |  |  |  |  |
| CAD | 0.796 (0.017) | 2.71 |  | 0.787 (0.017) | -1.13 |
| T2D | 0.727 (0.017) | 2.54 |  | 0.720 (0.017) | -0.96 |
| asthma | 0.565 (0.019) | 4.05 |  | 0.536 (0.019) | -5.13 |

Note: the performance of transPGS with EUR as auxiliary samples was compared to that with AFR as auxiliary samples; gain of prediction accuracy was calculated by comparing transPGS with PGS before transfer learning.

# Table S15. Estimated *R*^2^ or AUC of prediction models for the seven continuous phenotypes and three diseases in the UKB AFR population and the GERA AFR population before and after transfer learning with individual-level data.

| phenotype | EUR | | |  | CHI or EAS | |
| --- | --- | --- | --- | --- | --- | --- |
|  | transPGS (*se*) | | gain (%) |  | transPGS (*se*) | gain (%) |
| UKB (AFR) |  | |  |  |  |  |
| HDL | 0.192 (0.014) | | 15.66 |  | 0.165 (0.012) | -14.06 |
| LDL | 0.075 (0.024) | | 47.06 |  | 0.060 (0.015) | -20.00 |
| TC | 0.077 (0.021) | | 42.59 |  | 0.047 (0.012) | -38.96 |
| TG | 0.116 (0.031) | | 23.40 |  | 0.059 (0.006) | -49.14 |
| SBP | 0.180 (0.009) | | 11.11 |  | 0.165 (0.010) | -8.33 |
| DBP | 0.107 (0.010) | | 24.42 |  | 0.085 (0.008) | -20.56 |
| BMI | 0.088 (0.013) | | 12.82 |  | 0.087 (0.008) | -1.14 |
| CAD | 0.689 (0.006) | | 2.99 |  | 0.685 (0.006) | -0.58 |
| T2D | 0.644 (0.007) | | -3.88 |  | 0.637 (0.009) | -1.09 |
| asthma | 0.562 (0.005) | | 2.00 |  | 0.560 (0.007) | -0.36 |
| GERA (AFR) |  |  |  | |  |  |
| CAD | 0.763 (0.011) | | 1.46 |  | 0.762 (0.017) | -0.13 |
| T2D | 0.635 (0.012) | | 4.96 |  | 0.626 (0.019) | -1.42 |
| asthma | 0.597 (0.022) | | 2.23 |  | 0.593 (0.024) | -0.67 |

Note: the performance of transPGS with EUR as auxiliary samples was compared to that with CHI or EAS as auxiliary samples; gain of prediction accuracy was calculated by comparing transPGS with PGS before transfer learning.

# Table S16. Estimated *R*^2^ or AUC of prediction models for the seven continuous phenotypes and three diseases in the UKB CHI population and the GERA EAS population before and after transfer learning with summary-level data.

| phenotype | EUR | |  | AFR | |
| --- | --- | --- | --- | --- | --- |
|  | transPGS (*se*) | gain (%) |  | transPGS (*se*) | gain (%) |
| UKB (CHI) |  |  |  |  |  |
| HDL | 0.204 (0.029) | 17.22 |  | 0.195 (0.037) | 12.104 |
| LDL | 0.018 (0.009) | 13.96 |  | 0.016 (0.009) | -1.483 |
| TC | 0.028 (0.017) | 25.92 |  | 0.024 (0.017) | 7.343 |
| TG | 0.050 (0.021) | 5.37 |  | 0.048 (0.021) | 1.495 |
| SBP | 0.234 (0.028) | 16.17 |  | 0.204 (0.042) | 1.193 |
| DBP | 0.084 (0.025) | 8.47 |  | 0.077 (0.024) | -0.557 |
| BMI | 0.074 (0.025) | 17.42 |  | 0.059 (0.024) | -6.663 |
| GERA (EAS) |  |  |  |  |  |
| CAD | 0.785 (0.021) | 0.79 |  | / | / |
| T2D | 0.708 (0.019) | 0.52 |  | / | / |
| asthma | 0.540 (0.009) | 1.02 |  | 0.524 (0.009) | -2.032 |

Note: the performance of transPGS with EUR as auxiliary samples was compared to that with AFR as auxiliary samples; gain of prediction accuracy was calculated by comparing transPGS with PGS before transfer learning.

# Table S17. Estimated *R*^2^ or AUC of prediction models for the seven continuous phenotypes and one disease in the UKB AFR population and the GERA AFR population before and after transfer learning with summary-level data.

| phenotype | EUR | |  | CHI or EAS | |
| --- | --- | --- | --- | --- | --- |
|  | transPGS (*se*) | gain (%) |  | transPGS (*se*) | gain (%) |
| UKB (AFR) |  |  |  |  |  |
| HDL | 0.042 (0.011) | 28.58 |  | 0.034 (0.009) | 3.347 |
| LDL | 0.013 (0.008) | 9.39 |  | 0.012 (0.007) | -6.101 |
| TC | 0.020 (0.010) | 7.36 |  | 0.018 (0.010) | -4.813 |
| TG | 0.030 (0.008) | 2.36 |  | 0.029 (0.008) | -0.411 |
| SBP | 0.154 (0.013) | 3.53 |  | 0.149 (0.016) | 0.011 |
| DBP | 0.033 (0.007) | 1.15 |  | 0.033 (0.007) | -0.062 |
| BMI | 0.012 (0.006) | 0.03 |  | 0.011 (0.006) | -5.117 |
| GERA (AFR) |  |  |  |  |  |
| asthma | 0.529 (0.027) | 0.18 |  | 0.527 (0.029) | -0.175 |

Note: the performance of transPGS with EUR as auxiliary samples was compared to that with CHI or EAS as auxiliary samples; gain of prediction accuracy was calculated by comparing transPGS with PGS before transfer learning.

#
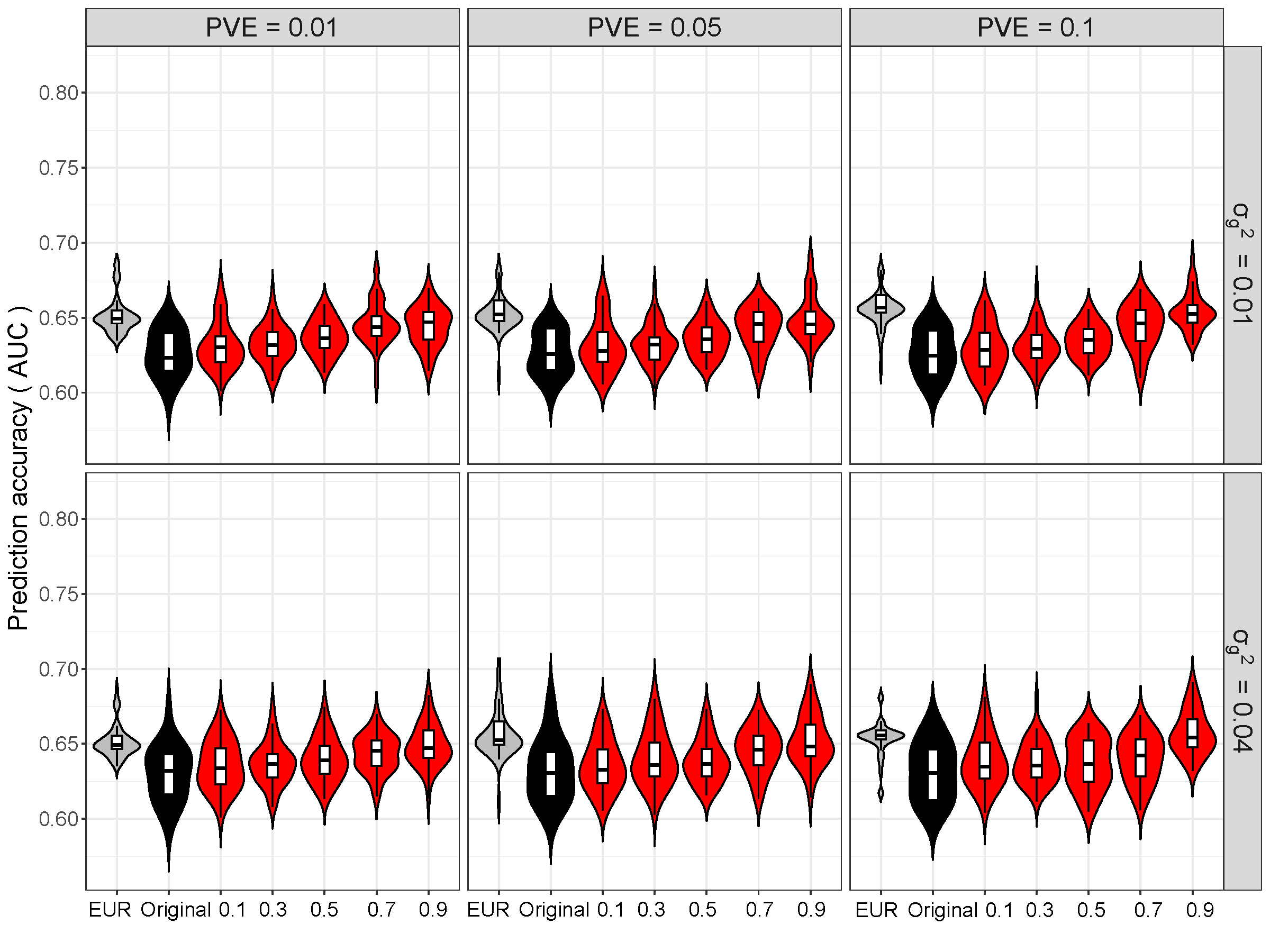
Figure S1. Prediction performance (AUC) of models in the simulations using a set of local SNPs for binary phenotypes in the target (AFR) and auxiliary (EUR) samples with various degrees of genetic overlap shared between the two populations. AUC was calculated before and after transfer learning, in which the shared information of the auxiliary population was incorporated into the target population. EUR: PGS model in the auxiliary samples; Original: PGS model in the target samples before transfer learning; *ω*=0.1, 0.3, 0.5, 0.7 and 0.9 indicates PGS model in the target samples after transfer learning (i.e., transPGS).


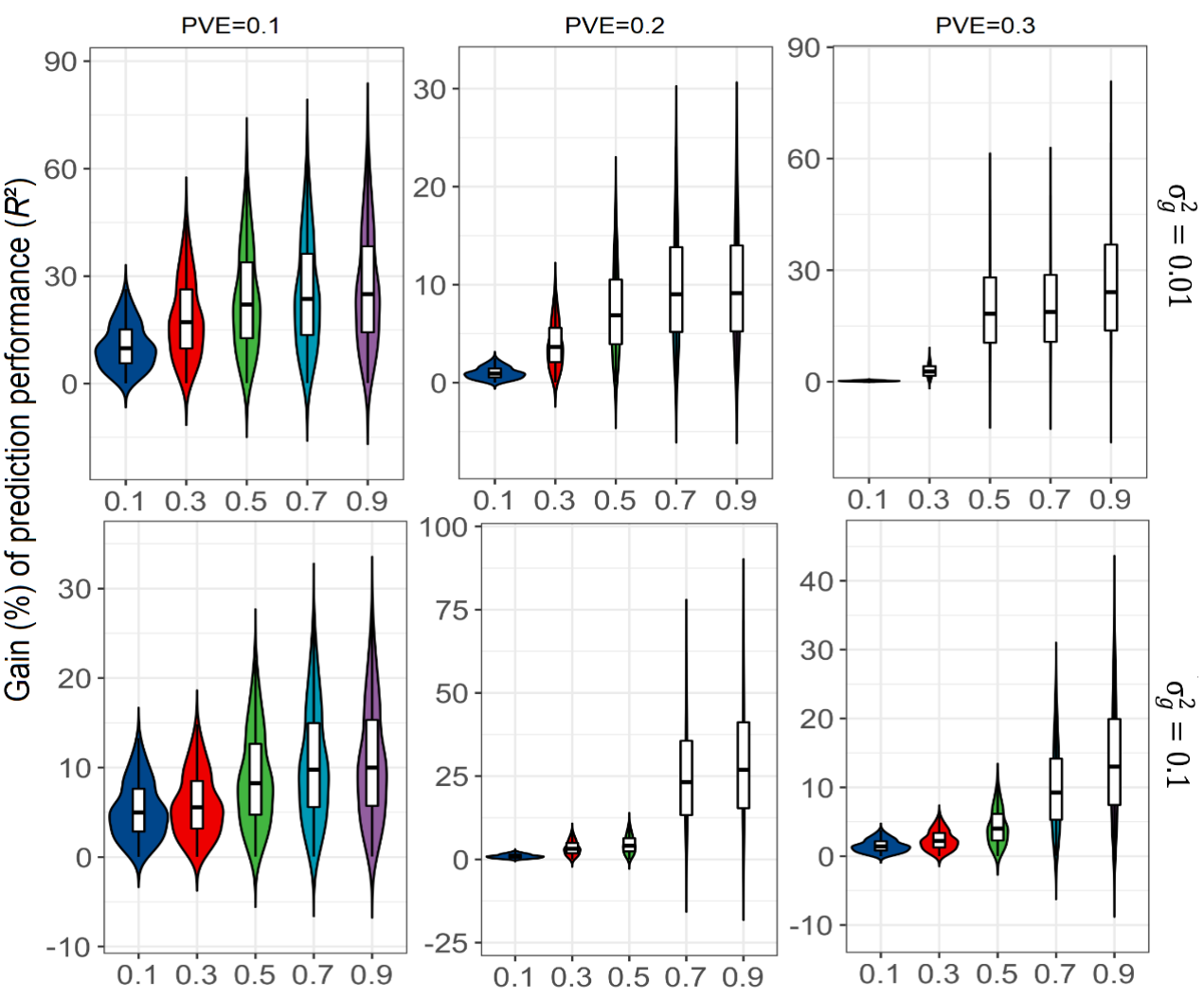


# Figure S2. Improved prediction accuracy (*R*^2^) of transPGS in the simulations using a set of local SNPs for continuous phenotype in the target (CHI) and auxiliary (EUR) samples with various degrees of genetic overlap shared between the two populations. *R*^2^ was calculated before and after transfer learning, in which the shared information of the auxiliary samples was incorporated into the target samples.


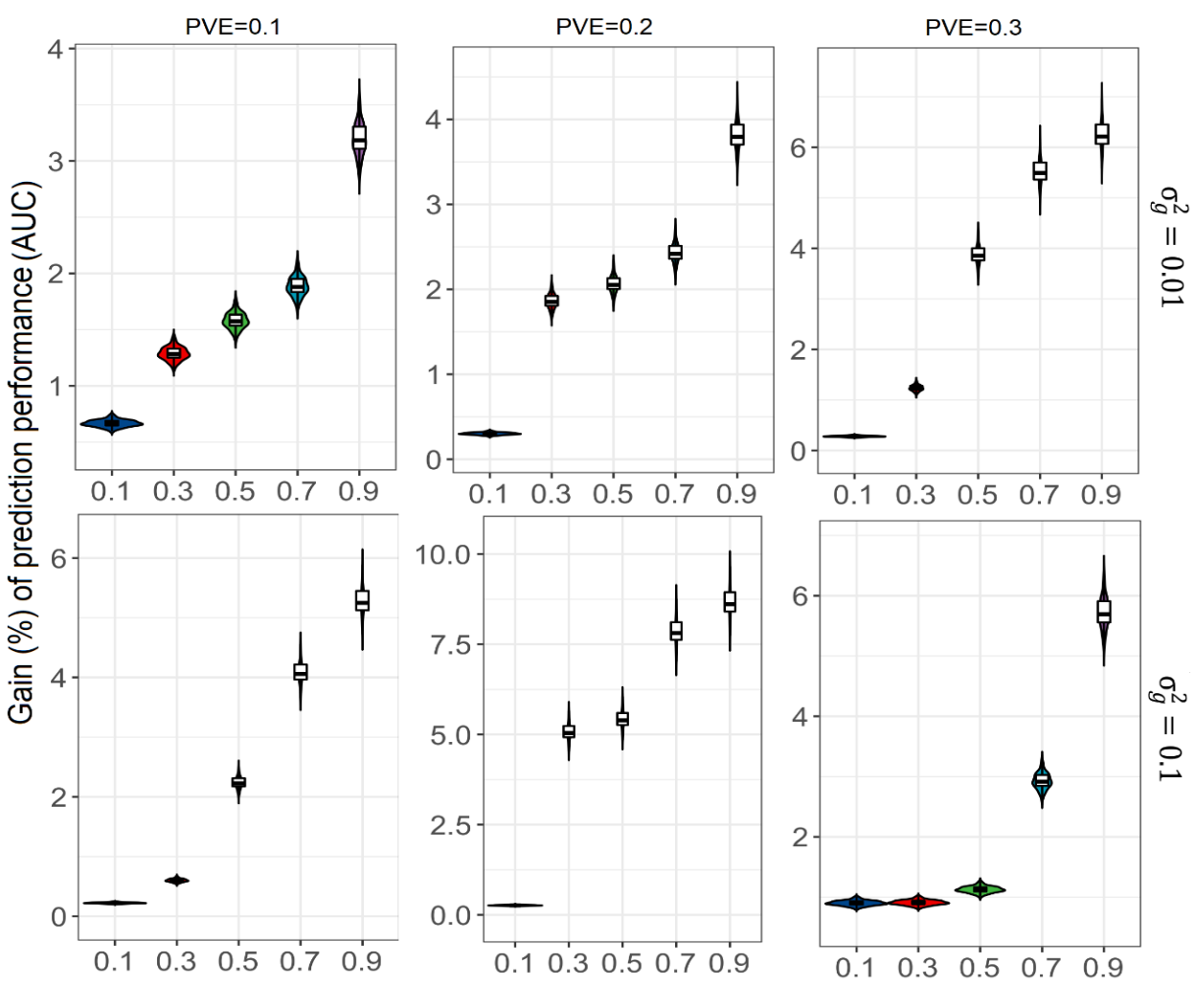


# Figure S3. Prediction performance (AUC) of models in the simulations using a set of local SNPs for binary phenotype in the target (CHI) and auxiliary (EUR) samples with various degrees of genetic overlap shared between the two populations. AUC was calculated before and after transfer learning, in which the shared information of the auxiliary population was incorporated into the target population.


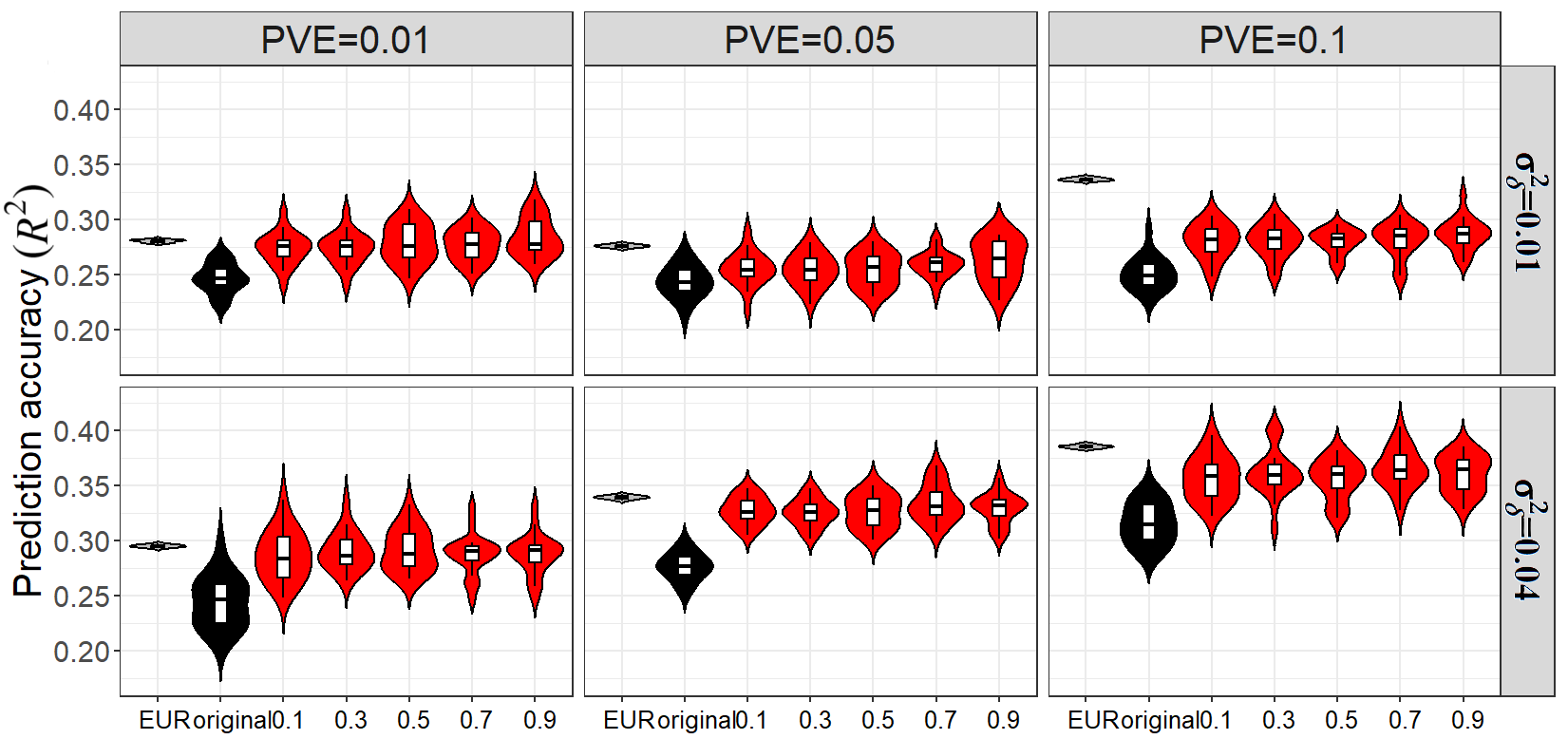


# Figure S4. Prediction performance (*R*^2^) of models in the simulations using genome-wide SNPs for continuous phenotypes in the target (AFR) and auxiliary (EUR) samples with various degrees of genetic overlap shared between the two populations. *R*^2^ was calculated before and after transfer learning, in which the shared information of the auxiliary population was incorporated into the target population. EUR: PGS model in the auxiliary samples; Original: PGS model in the target samples before transfer learning; *ω*=0.1, 0.3, 0.5, 0.7 and 0.9 indicates PGS model in the target samples after transfer learning (i.e., transPGS).


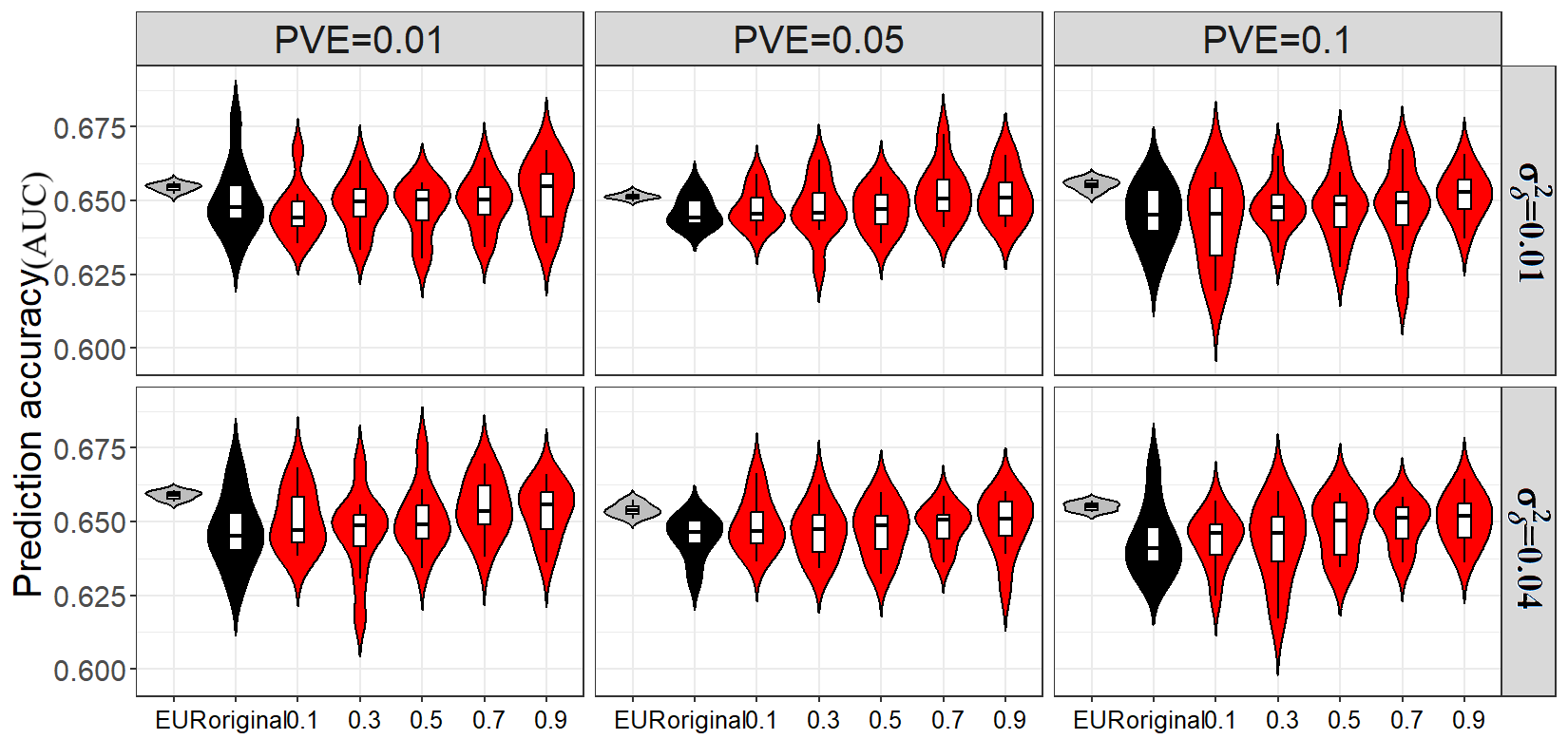


# Figure S5. Prediction performance (AUC) of models in the simulations using genome-wide SNPs for binary phenotypes in the target (AFR) and auxiliary (EUR) samples with various degrees of genetic overlap shared between the two populations. AUC was calculated before and after transfer learning, in which the shared information of the auxiliary population was incorporated into the target population. EUR: PGS model in the auxiliary samples; Original: PGS model in the target samples before transfer learning; *ω*=0.1, 0.3, 0.5, 0.7 and 0.9 indicates PGS model in the target samples after transfer learning (i.e., transPGS).


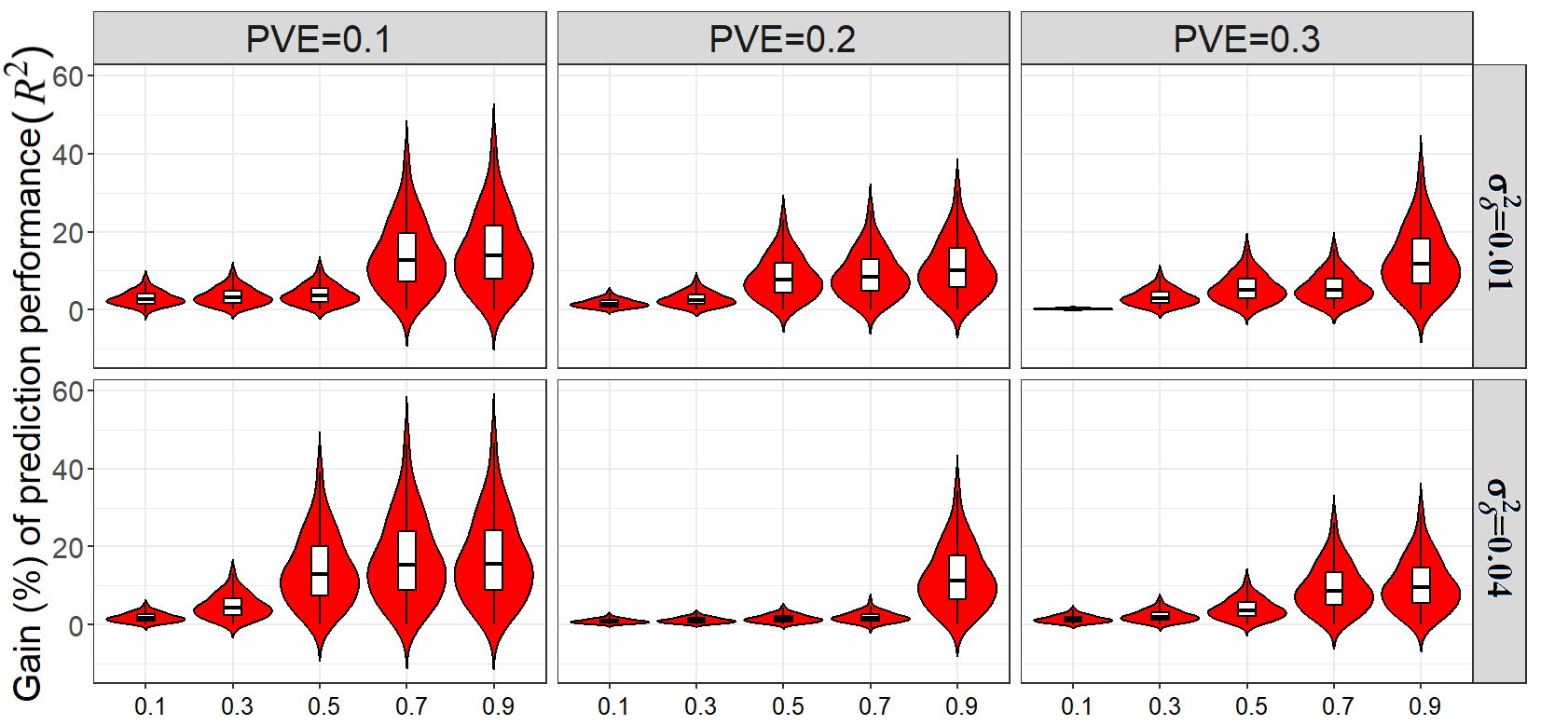


# Figure S6. Improved prediction accuracy (*R*^2^) of transPGS in the simulations using genome-wide SNPs for continuous phenotype in the target (CHI) and auxiliary (EUR) samples with various degrees of genetic overlap shared between the two populations. *R*^2^ was calculated before and after transfer learning, in which the shared information of the auxiliary samples was incorporated into the target samples.


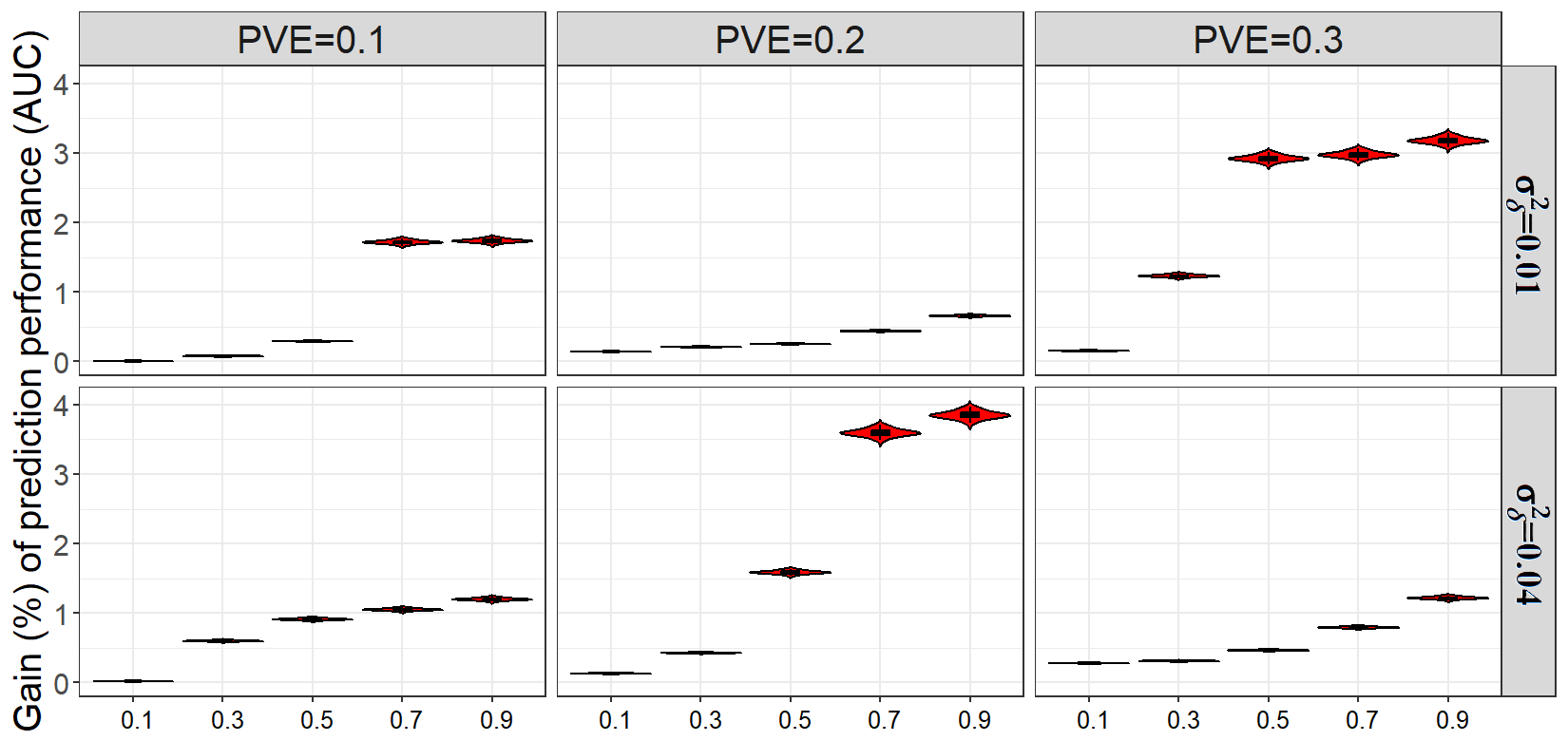


# Figure S7. Prediction performance (AUC) of models in the simulations using genome-wide SNPs for binary phenotype in the target (CHI) and auxiliary (EUR) samples with various degrees of genetic overlap shared between the two populations. AUC was calculated before and after transfer learning, in which the shared information of the auxiliary population was incorporated into the target population.


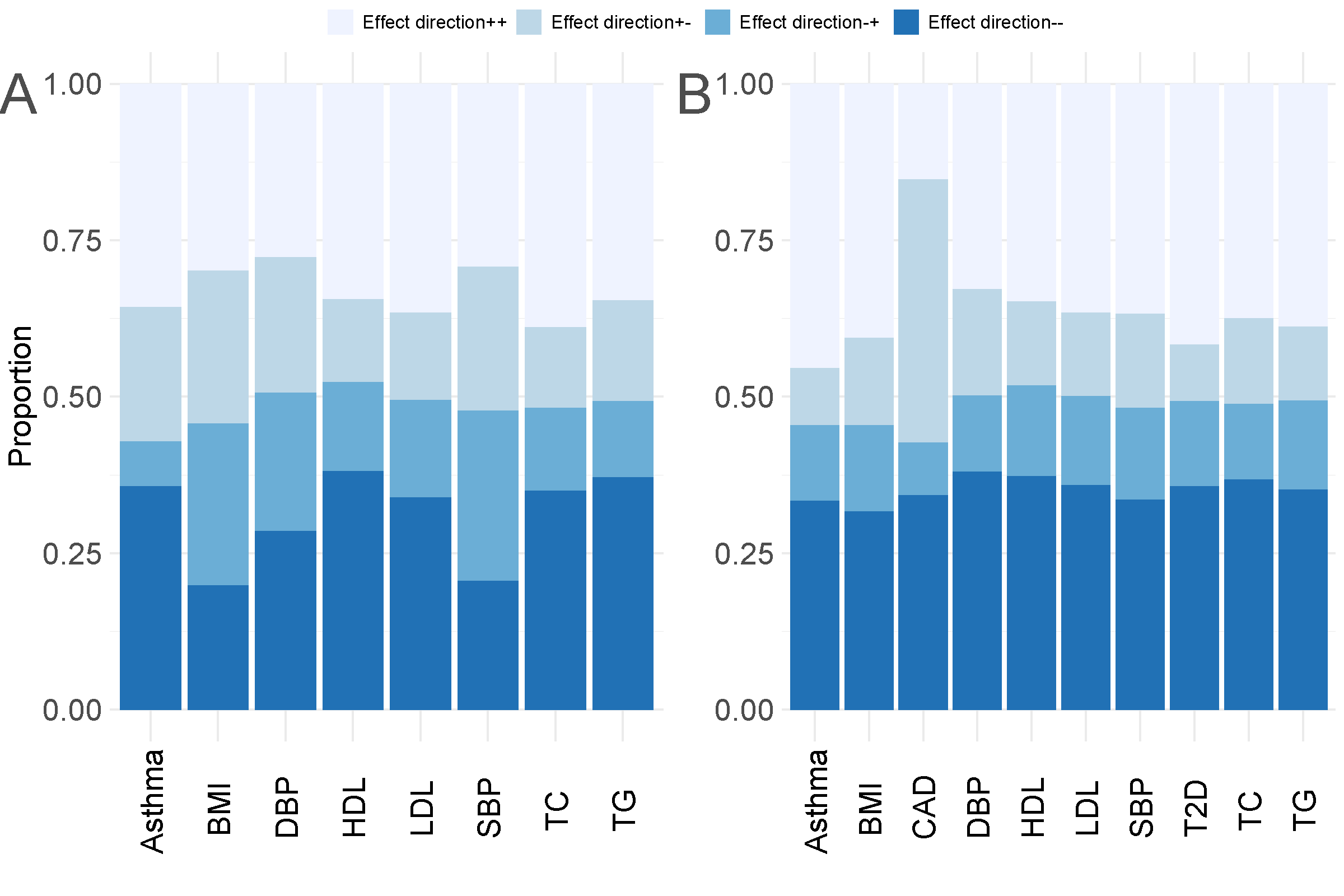


# Figure S8. (A) Proportion of SNP effect sizes with different effect directions between the AFR and EUR populations. (B) Proportion of SNP effect sizes with different effect directions between the EAS and EUR populations. ++ represents the proportion that SNPs had positive effect sizes in both populations; +- represents the proportion that SNPs had positive effect sizes in the EAS or AFR population while had negative effect sizes in the EUR population; -+ represents the proportion that SNPs had positive effect sizes in the EUR population while had negative effect sizes in the EAS or AFR population; -- represents the proportion that SNPs had negative effect sizes in both populations.


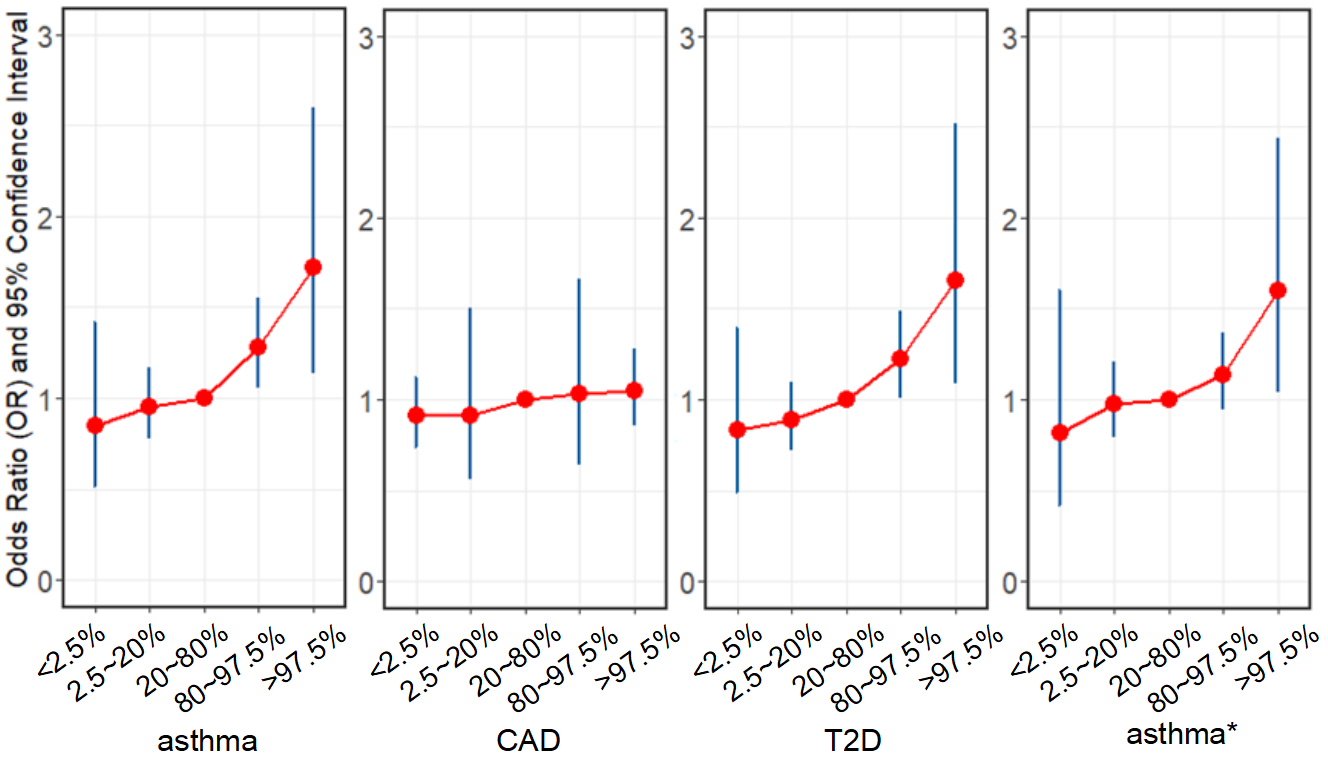


# Figure S9. Risk of occurring CAD, T2D and asthma in the UKB AFR cohort and risk of occurring asthma in the GERA AFR cohort for participants with the genetic risk being in the various intervals of the PGS distribution. The PGS was calculated by summary-level transPGS; the 20~80% PGS was used as the reference.

# References

1. Visscher PM, Wray NR, Zhang Q et al. 10 Years of GWAS Discovery: Biology, Function, and Translation, Am J Hum Genet 2017;101:5-22.

2. Zhu X, Stephens M. Bayesian large-scale multiple regression with summary statistics from genome-wide association studies, The Annals of Applied Statistics 2017;11:1561-1592.

3. The 1000 Genomes Project Consortium. A global reference for human genetic variation, Nature 2015;526:68-74.

4. Fan J, Liao Y, Liu H. An overview of the estimation of large covariance and precision matrices, The Econometrics Journal 2016;19:C1-C32.

5. Yang Y, Shi X, Jiao Y et al. CoMM-S2: a collaborative mixed model using summary statistics in transcriptome-wide association studies, Bioinformatics 2020;36:2009-2016.

6. Hoerl AE, Kennard RW. Ridge regression: Biased estimation for nonorthogonal problems, Technometrics 1970;12:55-67.

7. Tibshirani R. Regression Shrinkage and Selection via the Lasso, Journal of the Royal Statistical Society: Series B (Statistical Methodology) 1996;58:267-288.

8. Fu WJ. Penalized Regressions: The Bridge versus the Lasso, Journal of Computational and Graphical Statistics 1998;7:397-416.

9. Friedman JH, Hastie T, Tibshirani R. Regularization Paths for Generalized Linear Models via Coordinate Descent, Journal of Statistical Software 2010;33:1-22.

10. Zeng P, Dai J, Jin S et al. Aggregating multiple expression prediction models improves the power of transcriptome-wide association studies, Hum Mol Genet 2021;30:939-951.

11. Lu H, Zhang S, Jiang Z et al. Leveraging trans-ethnic genetic risk scores to improve association power for complex traits in underrepresented populations, Briefings in Bioinformatics 2023:bbad232.

12. Liu C, Donald B. Rubin, Ying Nian Wu. Parameter expansion to accelerate EM: The PX-EM algorithm, Biometrika 1998;85:755-770.

13. Lewandowski A, Liu C, Vander Wiel S. Parameter Expansion and Efficient Inference, Statistical Science 2010;25.

14. Yang C, Wan X, Lin X et al. CoMM: a collaborative mixed model to dissecting genetic contributions to complex traits by leveraging regulatory information, Bioinformatics 2019;35:1644-1652.

15. Wang T, Qiao J, Zhang S et al. Simultaneous test and estimation of total genetic effect in eQTL integrative analysis through mixed models, Briefings in Bioinformatics 2022;23:bbac038.

16. Zeng P, Zhou X. Non-parametric genetic prediction of complex traits with latent Dirichlet process regression models, Nature Communications 2017;8:456.

17. Liu C, Rubin DB, Wu YN. Parameter expansion to accelerate EM: The PX-EM algorithm, Biometrika 1998;85:755-770.

18. Chen H, Wang CL, Conomos MP et al. Control for Population Structure and Relatedness for Binary Traits in Genetic Association Studies via Logistic Mixed Models, American Journal of Human Genetics 2016;98:653-666.

19. Yang J, Lee S, Goddard M et al. GCTA: A Tool for Genome-wide Complex Trait Analysis, American Journal of Human Genetics 2011;88:76-82.

20. Lee SH, van der Werf JHJ. An efficient variance component approach implementing an average information REML suitable for combined LD and linkage mapping with a general complex pedigree, Genetics Selection Evolution 2006;38:25-43.

21. Zhou W, Nielsen JB, Fritsche LG et al. Efficiently controlling for case-control imbalance and sample relatedness in large-scale genetic association studies, Nature Genetics 2018;50:1335-1341.

22. Sun S, Zhu J, Mozaffari S et al. Heritability estimation and differential analysis of count data with generalized linear mixed models in genomic sequencing studies, Bioinformatics 2018;35:487-496.

23. Gilmour AR, Thompson R, Cullis BR. Average Information REML: An Efficient Algorithm for Variance Parameter Estimation in Linear Mixed Models, Biometrics 1995;51:1440-1450.

24. Breslow NE, Clayton DG. Approximate Inference in Generalized Linear Mixed Models, Journal of the American Statistical Association 1993;88:9-25.

25. Searle SR, Casella G, and McCulloch CE. Variance Components. New York: John Wiley & Sons, 1992.

26. Bycroft C, Freeman C, Petkova D et al. The UK Biobank resource with deep phenotyping and genomic data, Nature 2018;562:203-209.

27. Zeng P, Zhao Y, Qian C et al. Statistical analysis for genome-wide association study, J Biomed Res 2015;29:285-297.

28. Finucane HK, Bulik-Sullivan B, Gusev A et al. Partitioning heritability by functional annotation using genome-wide association summary statistics, Nature Genetics 2015;47:1228-1235.

29. Lu H, Qiao J, Shao Z et al. A comprehensive gene-centric pleiotropic association analysis for 14 psychiatric disorders with GWAS summary statistics, BMC Med 2021;19:314.

30. Lebenbaum M, Espin-Garcia O, Li Y et al. Development and validation of a population based risk algorithm for obesity: The Obesity Population Risk Tool (OPoRT), PLoS One 2018;13:e0191169.

31. Lu Y, Li G, Ferrari P et al. Associations of handgrip strength with morbidity and all-cause mortality of cardiometabolic multimorbidity, BMC Med 2022;20:191.

32. Warrington NM, Beaumont RN, Horikoshi M et al. Maternal and fetal genetic effects on birth weight and their relevance to cardio-metabolic risk factors, Nat Genet 2019;51:804-814.

33. Anstey DE, Booth JN, 3rd, Abdalla M et al. Predicted Atherosclerotic Cardiovascular Disease Risk and Masked Hypertension Among Blacks in the Jackson Heart Study, Circ Cardiovasc Qual Outcomes 2017;10:e003421.

34. Chen N, Fan F, Geng J et al. Evaluating the risk of hypertension in residents in primary care in Shanghai, China with machine learning algorithms, Front Public Health 2022;10:984621.

35. Zhao H, Zhang X, Xu Y et al. Predicting the Risk of Hypertension Based on Several Easy-to-Collect Risk Factors: A Machine Learning Method, Front Public Health 2021;9:619429.

36. Gallus S, Odone A, Lugo A et al. Overweight and obesity prevalence and determinants in Italy: an update to 2010, Eur J Nutr 2013;52:677-685.

37. Cameron AJ, Spence AC, Laws R et al. A Review of the Relationship Between Socioeconomic Position and the Early-Life Predictors of Obesity, Curr Obes Rep 2015;4:350-362.

38. Simmonds M, Llewellyn A, Owen CG et al. Predicting adult obesity from childhood obesity: a systematic review and meta-analysis, Obes Rev 2016;17:95-107.

39. Wang M, Zhou T, Song Y et al. Joint exposure to various ambient air pollutants and incident heart failure: a prospective analysis in UK Biobank, Eur Heart J 2021;42:1582-1591.

40. Banda Y, Kvale MN, Hoffmann TJ et al. Characterizing Race/Ethnicity and Genetic Ancestry for 100,000 Subjects in the Genetic Epidemiology Research on Adult Health and Aging (GERA) Cohort, Genetics 2015;200:1285-1295.

41. Kvale MN, Hesselson S, Hoffmann TJ et al. Genotyping Informatics and Quality Control for 100,000 Subjects in the Genetic Epidemiology Research on Adult Health and Aging (GERA) Cohort, Genetics 2015;200:1051-1060.

42. Gurdasani D, Carstensen T, Fatumo S et al. Uganda Genome Resource Enables Insights into Population History and Genomic Discovery in Africa, Cell 2019;179:984-1002.

43. Kanai M, Akiyama M, Takahashi A et al. Genetic analysis of quantitative traits in the Japanese population links cell types to complex human diseases, Nat Genet 2018;50:390-400.

44. Evangelou E, Warren HR, Mosen-Ansorena D et al. Genetic analysis of over 1 million people identifies 535 new loci associated with blood pressure traits, Nat Genet 2018;50:1412-1425.

45. Akiyama M, Okada Y, Kanai M et al. Genome-wide association study identifies 112 new loci for body mass index in the Japanese population, Nat Genet 2017;49:1458-1467.

46. Yengo L, Sidorenko J, Kemper KE et al. Meta-analysis of genome-wide association studies for height and body mass index in approximately 700000 individuals of European ancestry, Hum Mol Genet 2018;27:3641-3649.

47. Ishigaki K, Akiyama M, Kanai M et al. Large-scale genome-wide association study in a Japanese population identifies novel susceptibility loci across different diseases, Nat Genet 2020;52:669-679.

48. Myers RA, Scott NM, Gauderman WJ et al. Genome-wide interaction studies reveal sex-specific asthma risk alleles, Hum Mol Genet 2014;23:5251-5259.

49. Ferreira MAR, Mathur R, Vonk JM et al. Genetic Architectures of Childhood- and Adult-Onset Asthma Are Partly Distinct, Am J Hum Genet 2019;104:665-684.

50. Aragam KG, Jiang T, Goel A et al. Discovery and systematic characterization of risk variants and genes for coronary artery disease in over a million participants, Nat Genet 2022;54:1803-1815.

51. Spracklen CN, Horikoshi M, Kim YJ et al. Identification of type 2 diabetes loci in 433,540 East Asian individuals, Nature 2020;582:240-245.

52. Mahajan A, Taliun D, Thurner M et al. Fine-mapping type 2 diabetes loci to single-variant resolution using high-density imputation and islet-specific epigenome maps, Nat Genet 2018;50:1505-1513.

53. Qiao J, Shao Z, Wu Y et al. Detecting associated genes for complex traits shared across East Asian and European populations under the framework of composite null hypothesis testing, J Transl Med 2022;20:424.

54. Brown BC, Ye CJ, Price AL et al. Transethnic Genetic-Correlation Estimates from Summary Statistics, Am J Hum Genet 2016;99:76-88.

55. Zhang J, Zhang S, Qiao J et al. Similarity and diversity of genetic architecture for complex traits between East Asian and European populations, BMC Genomics 2023;24:314.

56. Qiao J, Wu Y, Zhang S et al. Evaluating significance of European-associated index SNPs in the East Asian population for 31 complex phenotypes, BMC Genomics 2023;24:324.

57. Purcell S, Neale B, Todd-Brown K et al. PLINK: a tool set for whole-genome association and population-based linkage analyses, Am J Hum Genet 2007;81:559-575.

58. Graham SE, Clarke SL, Wu K-HH et al. The power of genetic diversity in genome-wide association studies of lipids, Nature 2021;600:675-679.
